# Role of OAS gene family in COVID-19 induced heart failure

**DOI:** 10.1186/s12967-023-04058-x

**Published:** 2023-03-22

**Authors:** Li-Juan Gao, Zhong-Mei He, Yi-Ying Li, Rui-Rui Yang, Min Yan, Xuan Shang, Ji-Min Cao

**Affiliations:** Key Laboratory of Cellular Physiology, Department of Physiology, Ministry of Education, Shanxi Medical University, Taiyuan, 030001 Shanxi People’s Republic of China

**Keywords:** OAS gene family, COVID-19, SARS-CoV-2, Cardiomyocyte, Heart failure

## Abstract

**Background:**

COVID-19, the current global pandemic caused by SARS-CoV-2 infection, can damage the heart and lead to heart failure (HF) and even cardiac death. The 2',5'-oligoadenylate synthetase (OAS) gene family encode interferon (IFN)-induced antiviral proteins which is associated with the antiviral immune responses of COVID-19. While the potential association of OAS gene family with cardiac injury and failure in COVID-19 has not been determined.

**Methods:**

The expression levels and biological functions of OAS gene family in SARS-CoV-2 infected cardiomyocytes dataset (GSE150392) and HF dataset (GSE120852) were determined by comprehensive bioinformatic analysis and experimental validation. The associated microRNAs (miRNAs) were explored from Targetscan and GSE104150. The potential OAS gene family-regulatory chemicals or ingredients were predicted using Comparative Toxicogenomics Database (CTD) and SymMap database.

**Results:**

The OAS genes were highly expressed in both SARS-CoV-2 infected cardiomyocytes and failing hearts. The differentially expressed genes (DEGs) in the two datasets were enriched in both cardiovascular disease and COVID-19 related pathways. The miRNAs-target analysis indicated that 10 miRNAs could increase the expression of OAS genes. A variety of chemicals or ingredients were predicted regulating the expression of OAS gene family especially estradiol.

**Conclusion:**

OAS gene family is an important mediator of HF in COVID-19 and may serve as a potential therapeutic target for cardiac injury and HF in COVID-19.

**Supplementary Information:**

The online version contains supplementary material available at 10.1186/s12967-023-04058-x.

## Background

Coronavirus disease 2019 (COVID-19), caused by severe acute respiratory syndrome coronavirus 2 (SARS-CoV-2) infection [1], is a highly transmissible and pathogenic coronavirus emerged in late 2019 and has greatly threatened human health and public safety worldwide [2]. So far, the epidemic of COVID-19 has spread all over the world, as of 19 June 2022, over 536 million confirmed cases and over 6.3 million deaths have been reported globally [3]. Patients with COVID-19 exhibit a variety of symptoms, such as cough, fever, chest discomfort, and even respiratory distress syndrome in severe cases [4]. Those with comorbidities, older age and male sex tend to suffer from severe COVID-19 after infection [5, 6]. In addition to causing severe respiratory illness, COVID-19 can also damage multiple organs especially the heart, causing myocardial injury and HF [7, 8]. HF is the fourth most frequent complication of COVID‐19 [9], and is also the final outcome of most cardiovascular diseases. The mortality rate of HF is extremely high [10]. Therefore, it is necessary to investigate the regulatory mechanisms of HF in COVID‐19 in order to better control the worse cardiac consequences of this infectious disease.

In human genome, OAS gene family includes OAS1, OAS2, OAS3, and OASL [11, 12]. As an important immune regulator [13], OAS gene family participates in antiviral biological process and innate immune. Upon virus infection, OAS genes catalyze ATP into 2ʹ,5ʹ-linked oligomers of adenosine in the presence of double-stranded (ds) RNA. These oligomers then activate RNaseL [14, 15]. OAS proteins act as a sensor of dsRNA and can activate RNaseL. OAS/RNase L pathway plays a critical role in establishing antiviral state and halting viral infection [16, 17]. In this process, NOD-like proteins can interact with the OAS family to enhance the activity of RNaseL. The degraded RNA activates retinoic acid-inducible gene-I (RIG-1) [18, 19]. RIG-1 amplify production of IFN-α/β and activate NF-κB to produce inflammatory factors, leading to cell apoptosis [20]. In addition, the JAK-STAT pathway and IFN regulatory factor (IRF) family are involved in the regulation of this process [21]. However, it is unknown whether the OAS gene family actually plays a beneficial or harmful role in SARS-CoV-2-infected cardiomyocytes and COVID-19-induced cardiac injury and failure, as activations of antiviral genes do not necessarily produce a beneficial effect on the viral diseases such as COVID-19 [22, 23].

In the present study, we speculated that OAS gene family is an effective mediator for COVID-19 to worsen the cardiac function and cause HF. Numerous studies have found that untimely or overreacted antiviral responses may cause imbalance of the immune system, leading to inflammatory response or cytokine storm [24]. SARS-CoV-2 infects the respiratory cells and other cells including the cardiomyocytes, causes host cell apoptotic death by inducing inflammation and even cytokine storm [25, 26], thus may worsen the cardiac function and cause HF, one of the most common outcomes of COVID-19. Here, using extensive bioinformatic analyses and experimental validation, we found that the expressions of OAS genes were significantly upregulated in both the SARS-CoV-2-infected cardiomyocytes and in the failing hearts of COVID-19-free cases. These findings support our speculation and suggest that OAS gene family promotes the development of HF in COVID-19. Targeting OAS genes may be a potential therapeutic approach in treating COVID-19 associated HF.

## Materials and methods

### Data download

The original gene expression profiles of GSE150392, GSE120852, GSE147507, GSE 179850 and GSE104150 were downloaded from the National Center of Biotechnology Information—Gene Expression Omnibus (NCBI-GEO) database (https://www.ncbi.nlm.nih.gov/geo/), the largest, free and open accessing public gene expression database presently in the world.

GSE150392 is a dataset of mRNA expression in SARS-CoV-2 infected cardiomyocytes, including three groups of SARS-CoV-2 infected hiPSC-cardiomyocytes and three groups of hiPSC-cardiomyocytes with mock. The detailed information of GSE150392 dataset is shown in Table 1.

**Table 1:** Detail information of GSE150392

GEO accession	Organism	Platform	Contributor(s)	Sample	
GSE150392	Homo sapiens (Cell)	GPL18573	Sharma A, et al	SARS-CoV-2 Infected hiPSC- cardiomyocytes	hiPSC-cardiomyocytes with mock
				3	3

GSE120852 is a mRNA expression dataset of HF (COVID-19 free), including 5 non-failing (NF) left ventricles (LV), 5 NF right ventricles (RV), 5 LV tissues from subjects of LV failure, 5 RV tissues from LV failure, 5 LV tissues from biventricular failure (Bi-HF), and 5 RV tissues from Bi-HF. In the condition of LV failure, the mRNA expression profiles of LV and the RV were extracted and compared with the corresponding normal ventricular tissues (non-failing ventricles) to obtain the differential gene 1 (diff-1) and gene 2 (diff-2). In the condition of biventricular failure, the mRNA expression profiles of LV and RV were derived and compared with that of the corresponding normal ventricular tissues to obtain the differential gene 3 (diff-3) and gene 4 (diff-4). The detailed information of GSE120852 dataset is shown in Table 2.

**Table 2:** Detail information of GSE120852

GEO accession	Organism	Platform	Contributor(s)	Sample								
GSE120852	Homo sapiens (Heart tissue)	GPL11154	Luo X, et al		Diff-1		Diff-2		Diff-3		Diff-4	
				Condition	Non Failing (NF)	Left Ventricle Heart Failure (LV-HF)	Non Failing (NF)	Left Ventricle Heart Failure (LV-HF)	Non Failing (NF)	BiVentricular Heart Failure (Bi-HF)	Non Failing (NF)	BiVentricular Heart Failure (Bi-HF)
				Tissue	Left Ventricle (LV)	Left Ventricle (LV)	Right Ventricle (RV)	Right Ventricle (RV)	Left Ventricle (LV)	Left Ventricle (LV)	Right Ventricle (RV)	Right Ventricle (RV)
					5	5	5	5	5	5	5	5

GSE147507 and GSE179850 are mRNA expression databsets related with COVID-19. In GSE147507, we chose three groups of human lung epithelial (NHBE) cells treated with mock and three groups of NHBE cells treated with SARS-CoV-2, the detailed information is showed in Table 3. GSE179850 contains the blood mRNA information obtained from 16 blood samples of healthy controls and 31 blood samples of COVID-19 patients, details are displayed in Table 4.

**Table 3:** Detail information of GSE147507

GEO accession	Organism	Platform	Contributor(s)	Sample	
GSE147507	Homo sapiens (Cell)	GPL18573	Sharma A, et al	Mock treated NHBE cells	NHBE infected with SARS-CoV-2
				3	3

**Table 4:** Detail information of GSE179850

GEO accession	Organism	Platform	Contributor(s)	Sample	
GSE179850	Homo sapiens (Blood)	GPL28038	Ebihara T, et al	Healthy control	COVID-19 patient
				16	31

GSE104150 is a blood microRNA (miRNA) dataset of HF, including 7 blood samples of healthy controls and 9 blood samples of HF subjects. The detailed information of GSE104150 dataset is shown in Table 5.

**Table 5:** Detail information of GSE104150 (microRNA expression dataset)

GEO accession	Organism	Platform	Contributor(s)	Sample	
GSE104150	Homo sapiens (Blood)	GPL20712	Liu W, et al	Healthy control	Heart failure
				7	9

### Analysis of differentially expressed genes (DEGs) and miRNAs

The “limma” package [27] in the R language was used to analyze the data downloaded from NCBI-GEO. In the analysis, the data were processed by filtering low-expression genes and were standardized by normalizeBetweenArrays() function, and then linear model was constructed to obtain DEGs. Linear model was carried out using the lmFit() function. The P-value was adjusted by Benjamini–Hochberg method. P < 0.05 and |log2FC|> 1 were chosen as the cut-off criteria in the analyses of GSE150392 and GSE104150; P < 0.05 and |log2FC|> 0.8 were set as the cut-off criteria in GSE120852. The common DEGs in GSE120852 were generated and visualized using FunRich software (version 3.1.3.).

### Analysis of DEGs interactions and screening of hub genes

The protein–protein interaction (PPI) network of DEGs were determined using STRING (version 11.5, https://string-db.org/). STRING [28] is an online database for searching protein–protein interactions including direct physical interactions and indirect functional correlations between proteins. In the PPI analysis, the minimally required interaction score was set as medium confident 0.4. Then, results were used to screen hub genes using CytoHubba in Cytoscape (version 3.9.0.). CytoHubba [29] is a plugin in Cytoscape, it provides 12 topological analysis methods which can be used to explore important nodes in biological networks. We used Density of Maximum Neighborhood Component (DMNC), one of the 12 topological analysis methods, to search the top 30 hub genes.

### Creation of mouse heart failure model

8-week-old male C57BL/6 mice (22 − 25 g) were purchased from Sibeifu Co. (Beijing). All mice were maintained under specific-pathogen-free (SPF) conditions. HF was induced by transverse aortic constriction (TAC) for 8 weeks. TAC surgery was conducted as reported by Tavakoli et al. [30] and Wu et al. [31]. Pressure gradients between the proximal and distal sites of TAC were determined by doppler echocardiography. Heart weight/body weight (HW/BW) ratio, ejection fraction (EF), fractional shortening (FS), LV posterior wall thickness at end diastole (LVPWd), and LV posterior wall thickness at systole (LVPWs) were calculated.

### Cell culture and treatment

H9C2 cells (a rat myocardial cell line) were purchased from Shanghai Institutes for Biological Sciences, Chinese Academy of Sciences (Shanghai, China), and cultured in 6-well plates using DMEM with 10% fetal bovine serum and 1% penicillin/streptomycin at 37 ℃ in a humidified environment containing 5% CO_2_. Cells were cultured in serum-free DMEM medium for 12 h, then were challenged with PBS or angiotensin II (Ang II) for 48 h to induce cardiomyocyte hypertrophic injury. Cells were harvested for quantitative real-time PCR (qPCR).

### RNA isolation and qPCR

Total mRNA was extracted from mice failing heart tissues and H9C2 cells using TRIzol (Invitrogen, Carlsbad, CA), then was reverse-transcribed into cDNA according to the instructions of TaKaRa PrimeScript RT reagent Kit with gDNA Eraser (TaKaRa, Osaka, Japan), according to the manufacturer’s instruction. PCR amplifications were quantified according to the instructions of TaKaRa TB Green Premix Ex Taq II (TaKaRa, Osaka, Japan). The primer sequences for qPCR were designed by Sangon Biotech Co., Ltd (Shanghai, China). The expression data were normalized to the reference glyceraldehyde-3-phosphate dehydrogenase (GAPDH) and the mRNA levels were calculated using the 2^−∆∆Ct^ method. Primer sequences for qPCR are shown in Additional file 1: Table S1.

### Analyses of gene ontology (GO) and kyoto encyclopedia of genes and genomes (KEGG) pathway enrichment

GO and KEGG pathway of DEGs were analyzed using Metascape. Metascape [32] (http://metascape.org) is a public online database, it is designed to provide a comprehensive gene list annotation and analysis resource. GO is a gene function classification system to describe gene property. In the GO enrichment analysis, three aspects were analyzed, including biological processes (BP), cellular components (CC), and molecular functions (MF). KEGG pathway was used to analyze gene function, genomic information, and target relationship of pathways. P < 0.01 was chosen as the cut-off criteria.

### Correlation analysis of OAS genes with other genes

Correlation analysis is a statistical method used to evaluate the relationship between two variables. Pearson correlation analysis between two genes was performed and the criteria was set as P < 0.05.

### Analysis of miRNAs targeting the OAS gene family

The microRNAs (miRNAs) are short non-coding RNA molecules with 19 to 25 nucleotides in size and have the functions of degrading or blocking target mRNAs at the post-transcriptional level [33]. Targetscan [34] (http://www.targetscan.org/vert_72/) was used to identify the upstream miRNAs of OAS gene family.

### Prediction of chemicals and ingredients interacting with OAS gene family

Comparative Toxicogenomics Database (CTD) and SymMap database were used to analyze the chemicals or drugs which interact with the OAS gene family. CTD [35] (http://ctdbase.org/) is a powerful public database which integrates large amounts of data among chemicals, genes, functional phenotypes and diseases. It provides information on the associations of chemical-gene/protein, chemical-disease, and gene-disease, and thus helps to predict mechanistic hypotheses about the influence of environment on disease. In the CTD database, we chose chemicals with “interaction” > 3 as the cut-off criteria to perform further analysis. SymMap [36] database (http://www.symmap.org/) is an integrative database of Traditional Chinese Medicine (TCM) enhanced by symptom mapping. It contains six components, including herbs, TCM symptoms, modern medicine (MM) symptoms, ingredients, targets (or genes) and disease. Target function was used to analyze the ingredients that interact with the OAS gene family.

### Docking analysis of affinity between chemicals/ingredients and OAS gene family

Molecular docking approach was used to identify the affinities of chemicals/ingredients with OAS1, OAS2, OAS3, and OASL. The structures of the small molecules from PubChem database (https://pubchem.ncbi.nlm.nih.gov/) were downloaded, and Chem3D software was used to minimize the ligand molecular energy. The 3D structures of OAS genes were obtained from PDB database (https://www1.rcsb.org/) or UniProt database (https://www.uniprot.org/). AutoDockTools 1.5.6 software was used to find out the active pockets. Vina script was run to calculate the molecular binding energy, Vina ≤ − 7.0 kcal·mol^−1^ indicated strong binding of “ligand” with “receptor”. PyMOL software was used to display the results.

### Statistical analysis

GraphPad Prism 5.0 were used to performed statistical analysis. Data were presented as mean ± standard deviation (SD). Two-tail *t*-test was used for comparison of two groups. Statistical significance was set at P < 0.05.

## Results

### DEGs and hub genes derived from the GSE150392 dataset

**Fig. 1:**
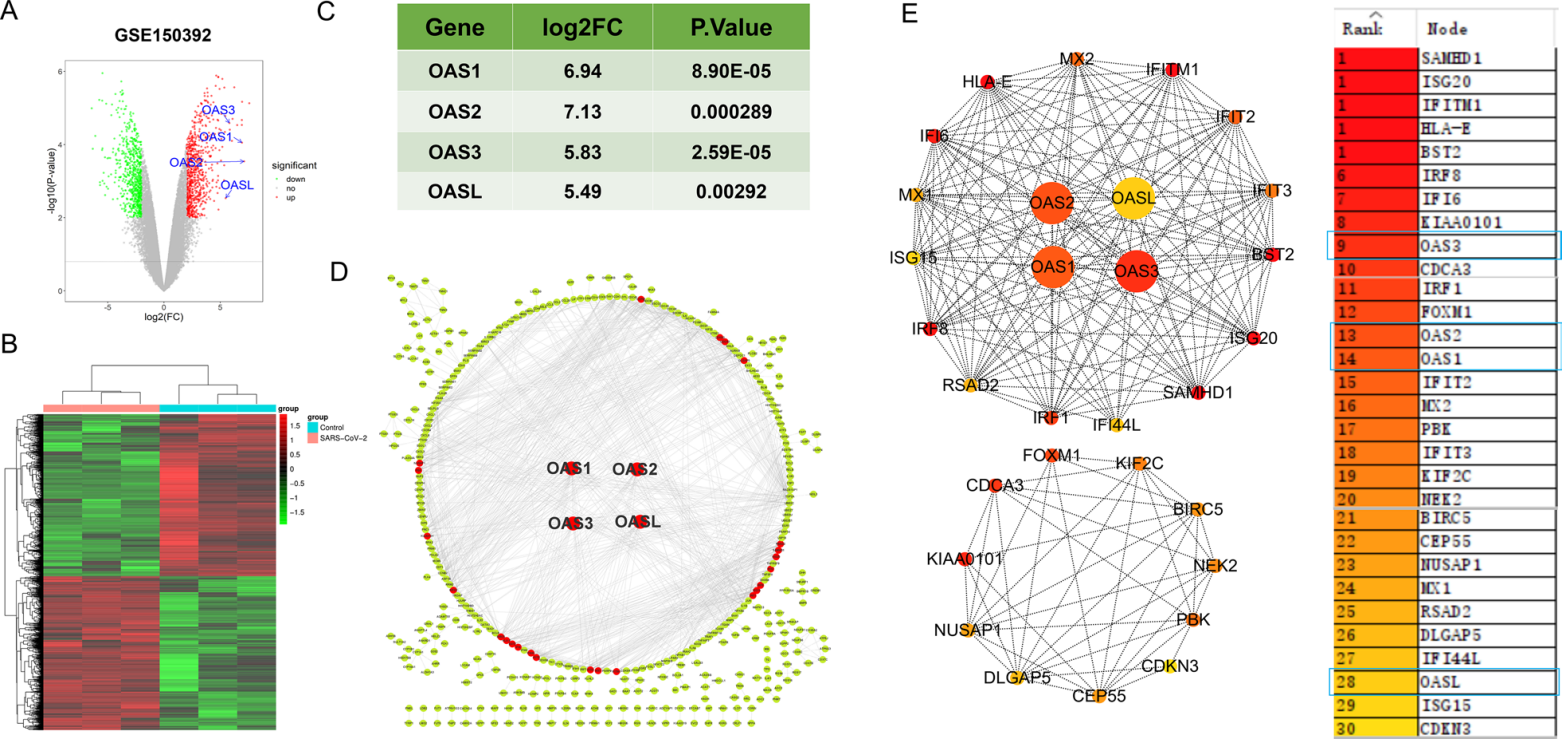
Data processing and hub genes screening in GSE150392. **A** Volcano plot of DEGs from GSE150392. X-axis indicates log2(FC) and Y-axis indicates − log10(P-value). Red and green dots represent upregulated and downregulated DEGs, respectively. Grey dots represent genes not differentially expressed. **B** Heatmap displaying all genes identified from GSE150392, with each column representing sample and each row representing gene. **C** OAS gene family expression in GSE150392. **D** PPI network of DEGs were showed with dots, red dot is hub gene as showed in E. **E** Top 30 hub genes calculated by cytohubba. Of these genes, OAS1, OAS2, OAS3, and OASL ranked 14, 13, 9, and 28, respectively

In GSE150392 (mRNA expression dataset of SARS-CoV-2 infected cardiomyocytes), a total of 1448 DEGs were screened out. Among them, 745 DEGs were up-expressed and 703 DEGs were down-expressed. Notably, OAS1, OAS2, OAS3, and OASL were all up-expressed in the cardiomyocytes with SARS-CoV-2 infection (Fig. 1A, B; Additional file 2), and their log2FCs were 6.94, 7.13, 5.83, and 5.49, respectively (Fig. 1C).

The protein–protein interactions among the DEGs were analyzed using STRING online database and results were presented using cytoscape (Fig. 1D). In this analysis, the algorithms of DMNC in the plugin cytoHubba was used to calculate the top 30 hub genes. OAS1, OAS2, OAS3, and OASL were ranked 14, 13, 9, and 28 among the top 30 hub genes, respectively (Fig. 1E).

### DEGs and hub genes derived from the GSE120852 dataset

**Fig. 2:**
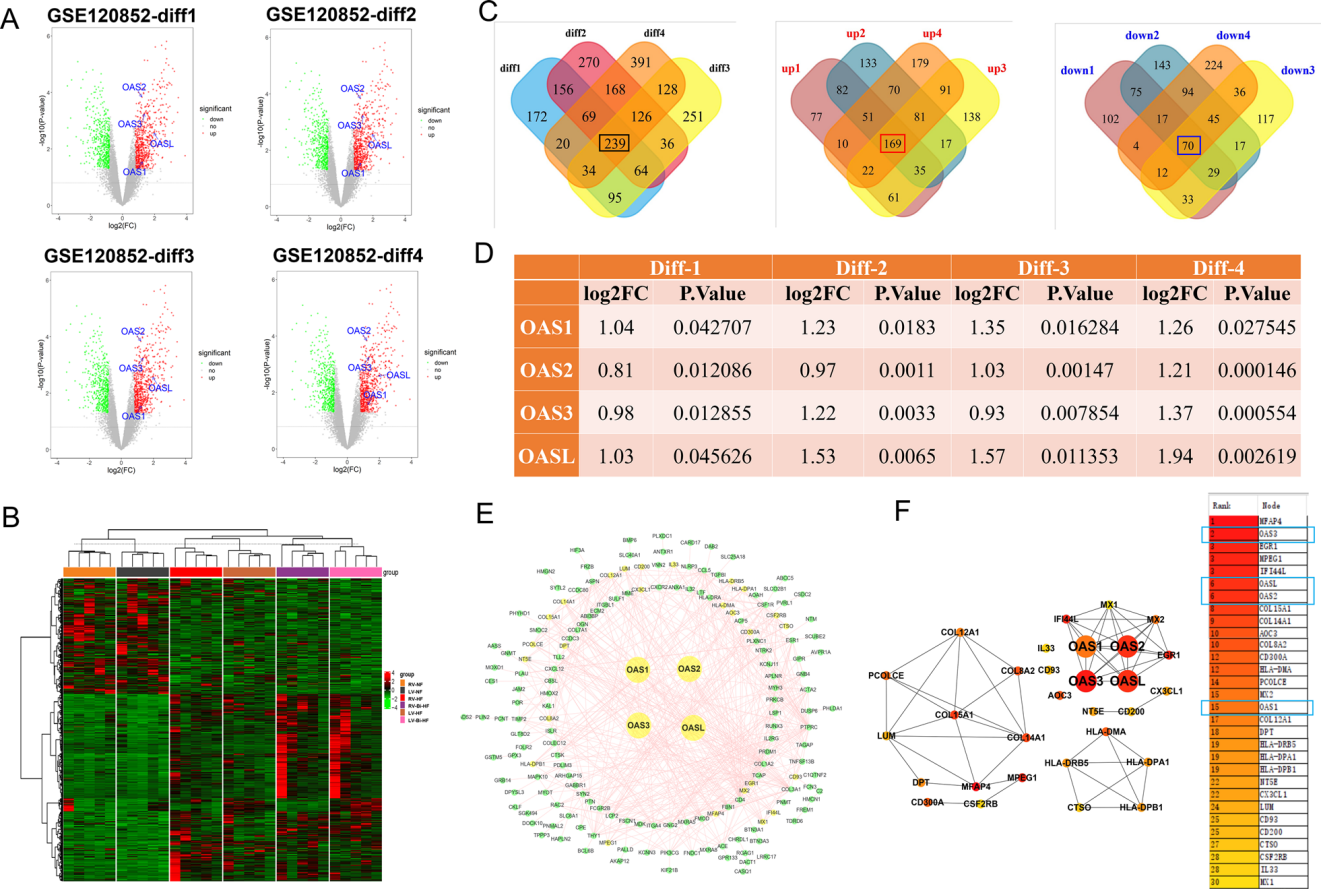
Data processing and hub genes screening in GSE120852. **A** Volcano plot of DEGs from GSE120852. X-axis indicates log2(FC) and the Y-axis indicates − log10(P-value). Red and green dots represent upregulated and downregulated DEGs, respectively. Grey dots represent genes not differentially expressed. Result of diff1 and diff3 came from left ventricle (LV) tissue, results of diff2 and diff4 were from right ventricle (RV) tissue, diff1 and diff2 are the results of comparing the left ventricle heart failure (LV-HF) and non-failing (NF) heart. Diff3 and diff4 are the results of comparing the biventricular heart failure (Bi-HF) and NF hearts. **B** Heatmap displaying all genes identified from GSE120852, with each column representing sample and each row indicating gene. RV-NF: right ventricular tissue, non-failing heart; LV-NF: LV tissue from non-failing hearts; RV-HF: failing right ventricular tissue; RV-Bi-HF: right ventricle tissue from biventricular failing hearts; LV-HF: failing LV tissue; LV-Bi-HF: left ventricular tissue from biventricular heart failure. **C** Venn diagram of DEGs in GSE120862. There were 239 common different genes in diff1- 4, 169 were up-expressed and 70 were down-expressed. **D** OAS gene family expression in GSE120862. **E** PPI network of common DEGs shown with dots. Yellow dots represent hub genes showing in F. **F** Top 30 hub genes calculated by cytohubba. Of these genes, OAS1, OAS2, OAS3, and OASL ranked 15, 6, 2, and 6, respectively

In GSE120852 (mRNA expression dataset of HF), four groups were included and their intersection genes (common DEGs) were selected to perform further analysis. Diff-1 indicated the comparison result of LV heart failure (LV-HF) vs. non-failing LV (LV-NF). As a result, 849 DEGs were screened out, 507 were up-expressed and 342 DEGs were down-expressed. Diff-2 was obtained from RV which reflected the RV mRNA difference between LV-HF and LV-NF. In the Diff-2 analysis, 1,128 DEGs were screened out, and among them, 638 were up-expressed and 490 DEGs were down-expressed. Diff-3 was obtained from LV by comparing biventricular heart failure (Bi-HF) and non-failing (NF) hearts. Total 973 DEGs were screened out, in which 614 were up-expressed and 359 DEGs were down-expressed. Diff-4 was gained from RV by comparing Bi-HF and NF hearts, total 1,175 DEGs were screened out, 673 were up-expressed and 502 DEGs were down-expressed (Fig. 2A, B; Additional file 3). Total 239 common DEGs were found in the above four groups of DEGs, and 169 were up-expressed and 70 were down-expressed (Fig. 2C). Of note, OAS1, OAS2, OAS3 and OASL were all highly expressed in HF. In diff-1, the log2FCs of OAS1, OAS2, OAS3, and OASL were 1.04, 0.81, 0.98, and 1.03, respectively. In diff-2, the log2FCs of OAS1, OAS2, OAS3 and OASL were 1.23, 0.97, 1.22, and 1.53, respectively. In diff-3, the log2FCs of OAS1, OAS2, OAS3 and OASL were 1.35, 1.03, 0.93, and 1.57, respectively. In diff-4, the log2FCs of OAS1, OAS2, OAS3 and OASL were 1.26, 1.21, 1.37 and 1.94, respectively (Fig. 2D).

SRTING online software was used to explore the relationships among the 239 common DEGs, and results were shown via cytoscape (Fig. 2E). The algorithm of DMNC in plugin cytoHubba was used to calculate the top 30 hub genes. To our speculation, OAS1, OAS2, OAS3, and OASL were all among the top 30 hub genes, and they were ranked 15, 6, 6, and 2, respectively (Fig. 2F).

### Experimental validation of high mRNA expressions of OAS genes

**Fig. 3:**
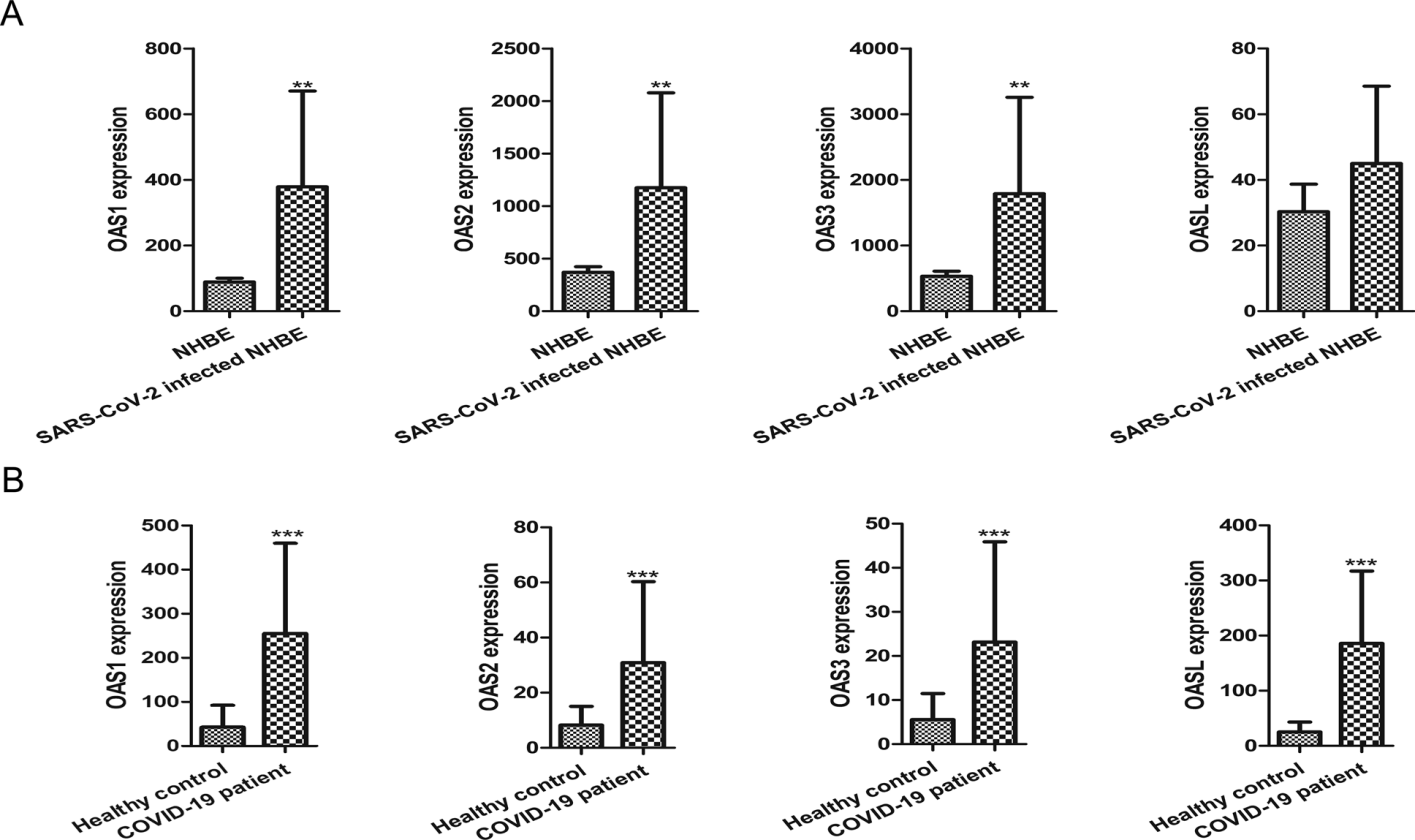
Experimental verification on the expression of OAS gene family in SARS-CoV-2 infected NHBE and COVID-19 blood. **A** OAS1, OAS2, OAS3, and OASL expression in GSE147507. NHBE: Mock treated primary human lung epithelium NHBE cells, n = 3; SARS-CoV-2 infected NHBE, n = 3. **B** OAS1, OAS2, OAS3, and OASL expression in GSE179850; Healthy control, n = 16; COVID-19 patient, n = 31. * P < 0.05, ** P < 0.01, *** P < 0.001

**Fig. 4:**
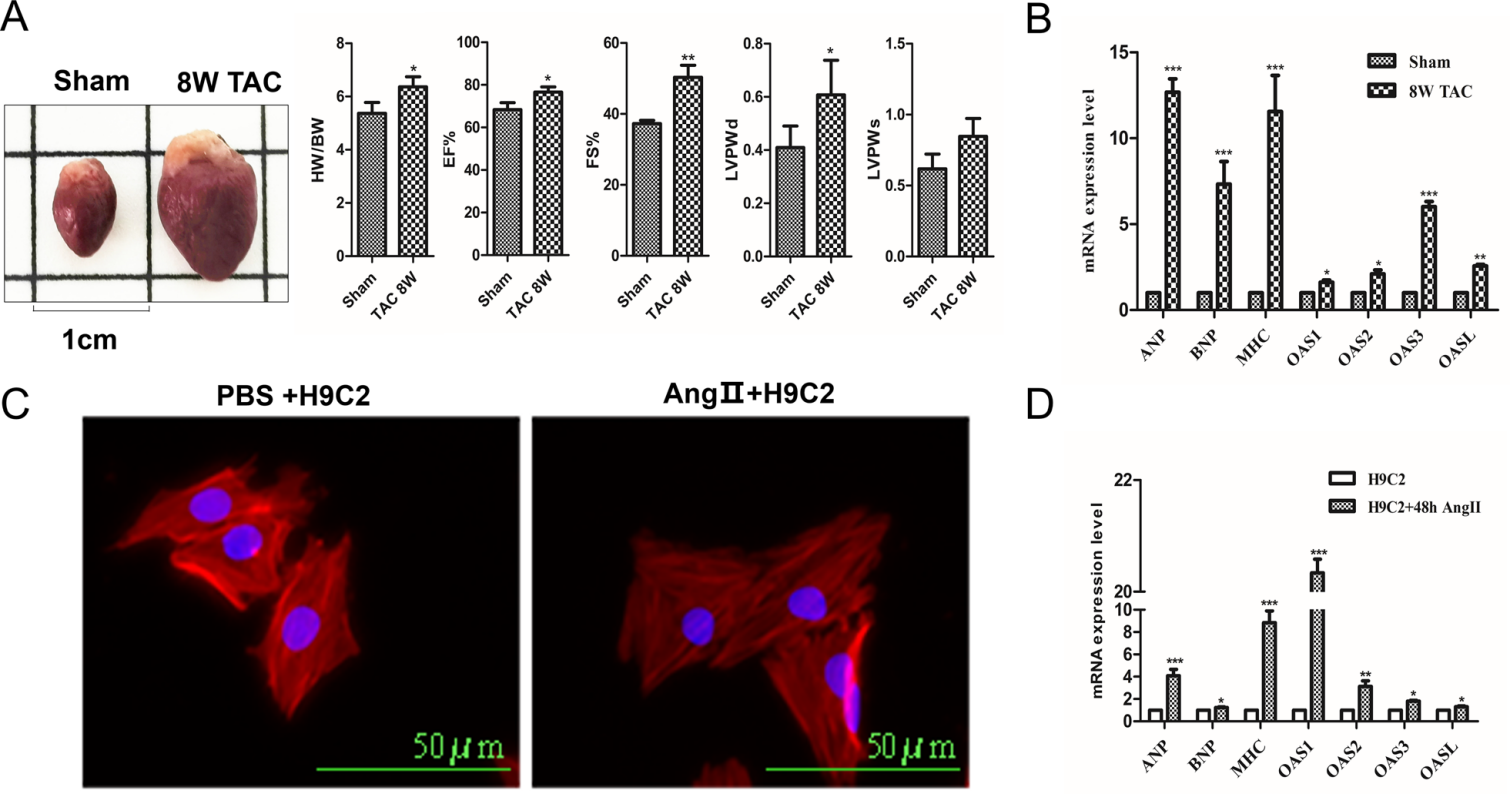
Experimental verification the expression of OAS gene family in HF. **A** Representative heart sizes from each group (left of upper row) and M-mode echocardiography of heart (right of upper row), measurement of HW/BW, EF%, FS%, LVPWD, and LVPWS, n = 4 samples for each group. **B** qPCR analysis of ANP, BNP, β-MHC, OAS1, OAS2, OAS3 and OASL mRNA levels in sham and 8W TAC hearts. Data were normalized to the GAPDH expression, n = 4 samples for each group. ANP, BNP, and β-MHC are HF markers. **C** H9C2 cells treated with PBS and Ang II (1 μM) for 48 h and performed to determine cell size with phalloidin and DAPI, n = 3 samples for each group. **D** qPCR analysis of ANP, BNP, β-MHC, OAS1, OAS2, OAS3 and OASL mRNA levels in PBS and 1 μM Ang II-treated H9C2 cells. Data were normalized to the GAPDH expression, n = 3 samples for each group. ANP, BNP, and β-MHC are HF markers. * P < 0.05, ** P < 0.01, *** P < 0.001

To verify the expression of OAS genes shown in Fig. 1, we further analyzed the GSE147507 dataset (SARS-CoV-2 infected NHBE) and the GSE179850 dataset (blood sample of COVID-19 patients) to evaluate the expression of OAS gene family in alternative tissues of COVID-19 patients. Results demonstrated that OAS1, OAS2, OAS3, and OASL were all highly expressed in SARS-CoV-2 infected NHBE (Fig. 3A) (OAS 1 − 3, P < 0.01) and in the blood leucocytes of COVID-19 patients (Fig. 3B) (all P < 0.001). To further validate the expression of OAS genes shown in Fig. 2, we performed qPCR experiments to measure the cardiac expression of OAS gene family in 8-week TAC mice (n = 4 samples for each group) and Ang II-challenged H9C2 cells (n = 3 samples for each group). Result showed that the mRNA levels of atrial natriuretic peptide (ANP), brain natriuretic peptide (BNP), and myosin heavy chain β (β-MHC) were all significantly increased in the cardiac tissues of TAC mice (Fig. 4A, B) and Ang II-challenged H9C2 cells (Fig. 4C, B), and the mRNA levels of OAS1, OAS2, OAS3, and OASL were also significantly elevated in the failing hearts of TAC mice (Fig. 4B) and Ang II-treated H9C2 cells (Fig. 4D). These results consistently indicate that OAS gene family was highly expressed in both SARS-CoV-2 infected cardiomyocytes and failing hearts.

### GO and KEGG pathway analyses revealing the intersecting signaling between COVID-19 and HF

The biological function enrichment analyses of DEGs in GSE150392 and GSE120852 datasets were performed using Metascape.

**Fig. 5:**
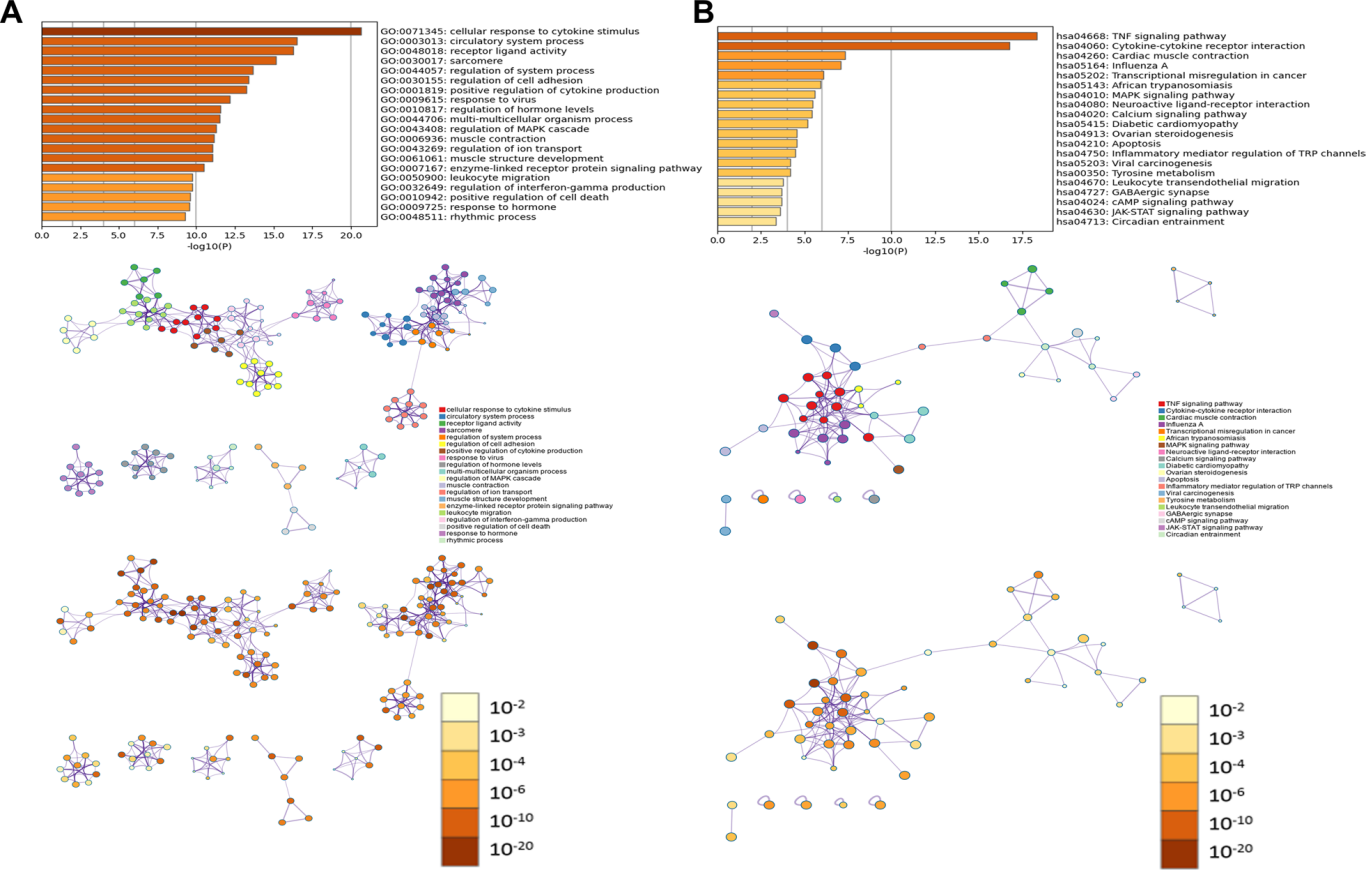
GO and KEGG pathway enrichment analysis of DEGs in GSE150392 by Metascape. **A** GO enrichment analysis. **B** KEGG pathway enrichment analysis

Results of GO analysis from GSE150392 are presented in Fig. 5A. The DEGs were significantly enriched in 20 GO terms. Results of KEGG pathway analysis from GSE150392 were displayed in Fig. 5B, which included 20 KEGG pathways. The top 10 GO terms and KEGG pathways were shown in Table 6 and details were listed in Additional file 1: Table S4 and Table S5.

**Table 6:** Top 10 GO terms and KEGG pathways of GSE150392

GO term			KEGG pathway		
Term	Description	LogP	Term	Description	LogP
GO:0071345	Cellular response to cytokine stimulus	− 20.64	hsa04668	TNF signaling pathway	− 18.34
GO:0003013	Circulatory system process	− 16.52	hsa04060	Cytokine-cytokine receptor interaction	− 16.78
GO:0048018	Receptor ligand activity	− 16.29	hsa04260	Cardiac muscle contraction	− 7.33
GO:0030017	Sarcomere	− 15.15	hsa05164	Influenza A	− 7.10
GO:0044057	Regulation of system process	− 13.66	hsa05202	Transcriptional misregulation in cancer	− 6.11
GO:0030155	Regulation of cell adhesion	− 13.39	hsa05143	African trypanosomiasis	− 5.92
GO:0001819	Positive regulation of cytokine production	− 13.24	hsa04010	MAPK signaling pathway	− 5.61
GO:0009615	Response to virus	− 12.17	hsa04080	Neuroactive ligand-receptor interaction	− 5.46
GO:0010817	Regulation of hormone levels	− 11.56	hsa04020	Calcium signaling pathway	− 5.42
GO:0044706	Multi-multicellular organism process	− 11.50	hsa05415	Diabetic cardiomyopathy	− 5.19

**Fig. 6:**
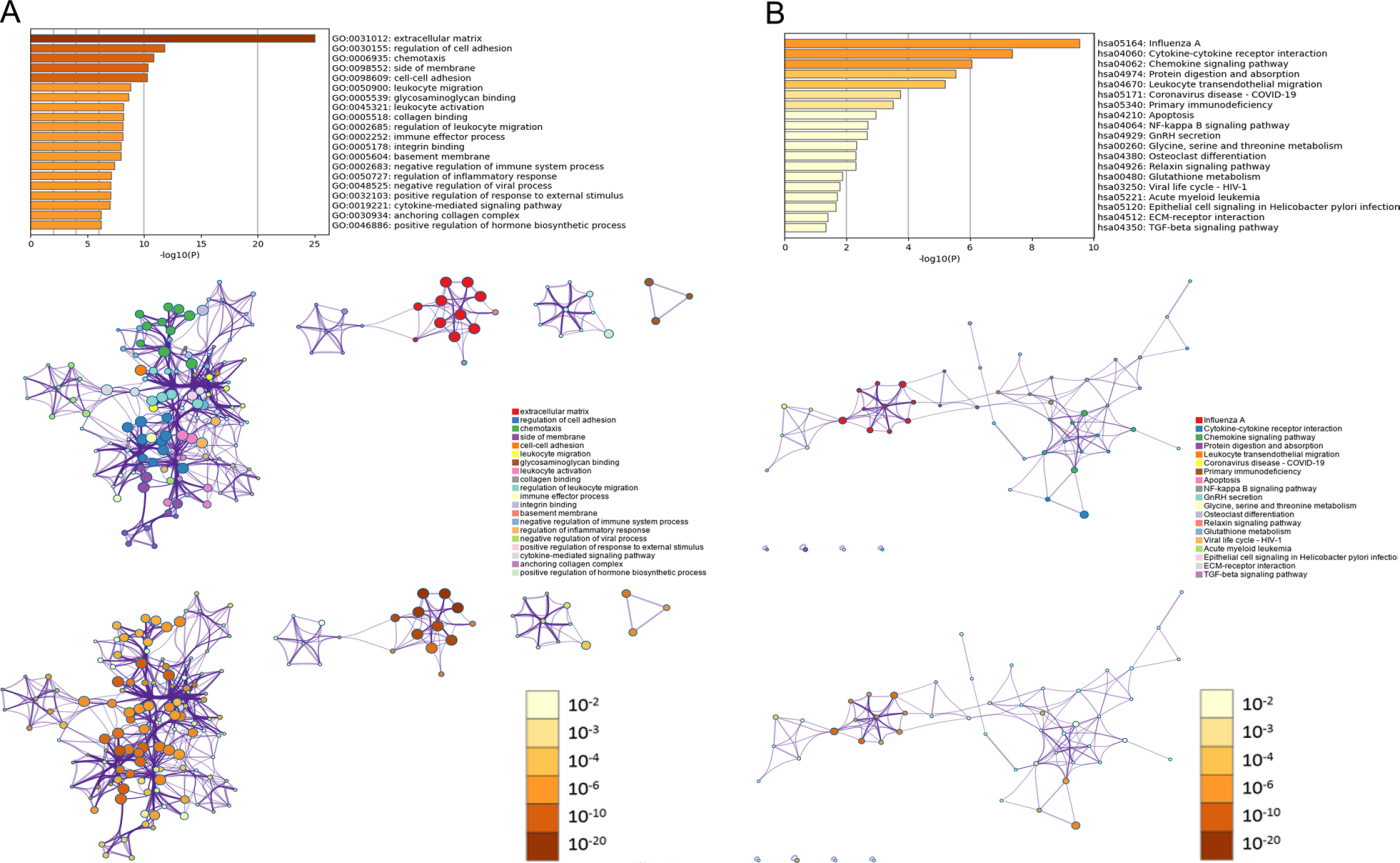
GO and KEGG pathway enrichment analysis of common DEGs in GSE120852 by Metascape. **A** GO enrichment analysis. **B** KEGG pathway enrichment analysis

Results of GO analysis from GSE120852 were displayed in Fig. 6A. The DEGs of this analysis were enriched in 20 GO terms. Results of KEGG pathway analysis from GSE120852 were displayed in Fig. 6B. The DEGs of this analysis included 19 KEGG pathways. The top 10 GO terms and KEGG pathways were shown in Table 7 and details were listed in Additional file 1: Table S6 and Table S7.

**Table 7:** Top 10 GO terms and KEGG pathways of GSE120852

GO term			KEGG pathway		
Term	Description	LogP	Term	Description	LogP
GO:0031012	Extracellular matrix	− 25.03	hsa05164	Influenza A	− 9.54
GO:0030155	Regulation of cell adhesion	− 11.77	hsa04060	Cytokine-cytokine receptor interaction	− 7.35
GO:0006935	Chemotaxis	− 10.82	hsa04062	Chemokine signaling pathway	− 6.05
GO:0098552	Side of membrane	− 10.31	hsa04974	Protein digestion and absorption	− 5.52
GO:0098609	Cell–cell adhesion	− 10.29	hsa04670	Leukocyte transendothelial migration	− 5.19
GO:0050900	Leukocyte migration	− 8.80	hsa05171	Coronavirus disease—COVID-19	− 3.73
GO:0005539	Glycosaminoglycan binding	− 8.65	hsa05340	Primary immunodeficiency	− 3.51
GO:0045321	Leukocyte activation	− 8.20	hsa04210	Apoptosis	− 2.94
GO:0005518	Collagen binding	− 8.17	hsa04064	NF-kappa B signaling pathway	− 2.69
GO:0002685	Regulation of leukocyte migration	− 8.14	hsa04929	GnRH secretion	− 2.66

Results of GO:0003013 (circulatory system process) and hsa04260 (cardiac muscle contraction) in GSE150392 analysis were related to cardiac function in SARS-CoV-2 infected cardiomyocytes. Some results from GSE120852, such as GO:0050727 (regulation of inflammatory response) and hsa05171 (COVID-19), were related to COVID-19. Above results suggest a relationship between COVID-19 and HF. In addition, some results, including GO:0050900 (BP: leukocyte migration), hsa04060 (Cytokine-cytokine receptor interaction), and hsa04210 (apoptosis), etc., were common results of GSE150392 and GSE120852, suggesting that the processes of HF and COVID-19 employ the same biological pathways to some extents.

### Correlated genes of OAS gene family

**Fig. 7:**
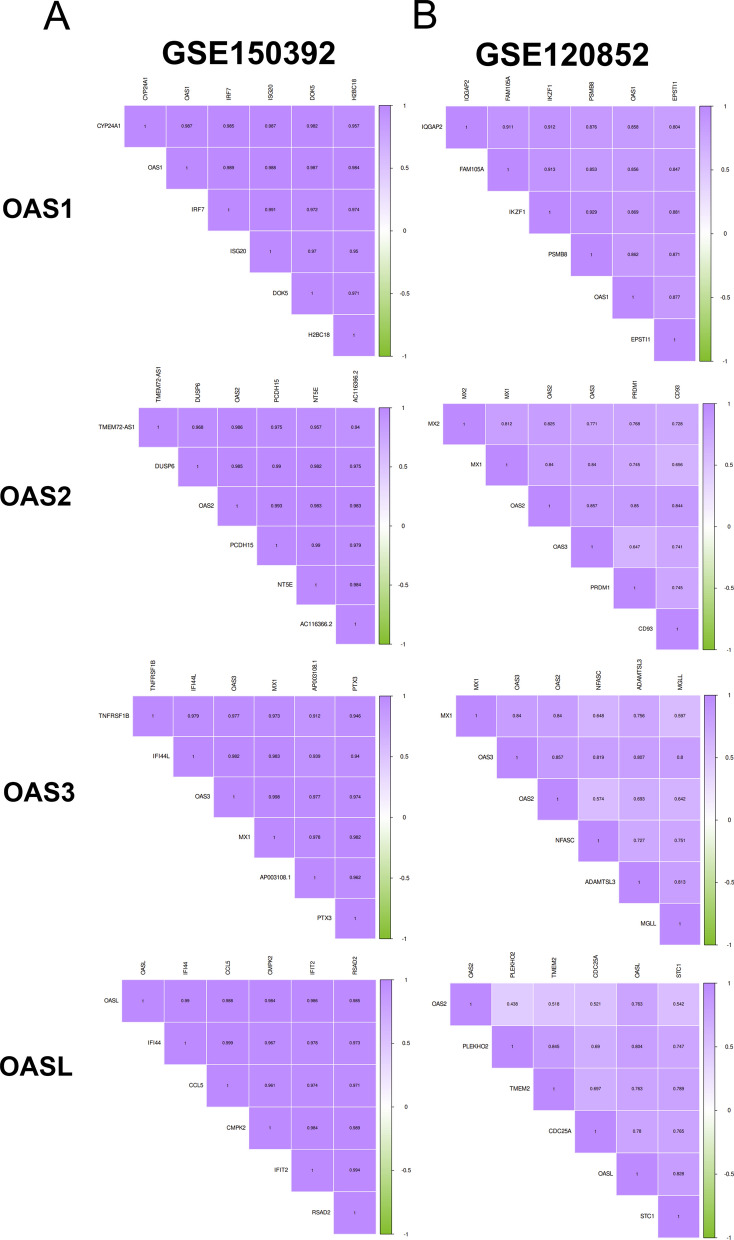
Correlated genes of OAS gene family. **A** Correlated genes of OAS gene family in GSE150392. **B** Correlated genes of OAS gene family in GSE120852

We further explored the top 5 genes which were correlated with each of the OAS genes. Correlated genes of OAS family in COVID-19 dataset (GSE150392) were revealed in Fig. 7A and Additional file 1: Table S8. Related genes of OAS family in HF dataset (GSE120852) were displayed in Fig. 7B and Additional file 1: Table S9. Results showed that IRF7, PCDH15, MX1, and IFI44 were the most relevant correlated genes of OAS1, OAS2, OAS3, and OASL in GSE150392, respectively. EPSTI1, OAS3, OAS2, and STC1 were the most interrelated genes of OAS1, OAS2, OAS3, and OASL in GSE120852, respectively.

### The miRNAs regulating the expression of OAS gene family

**Fig. 8:**
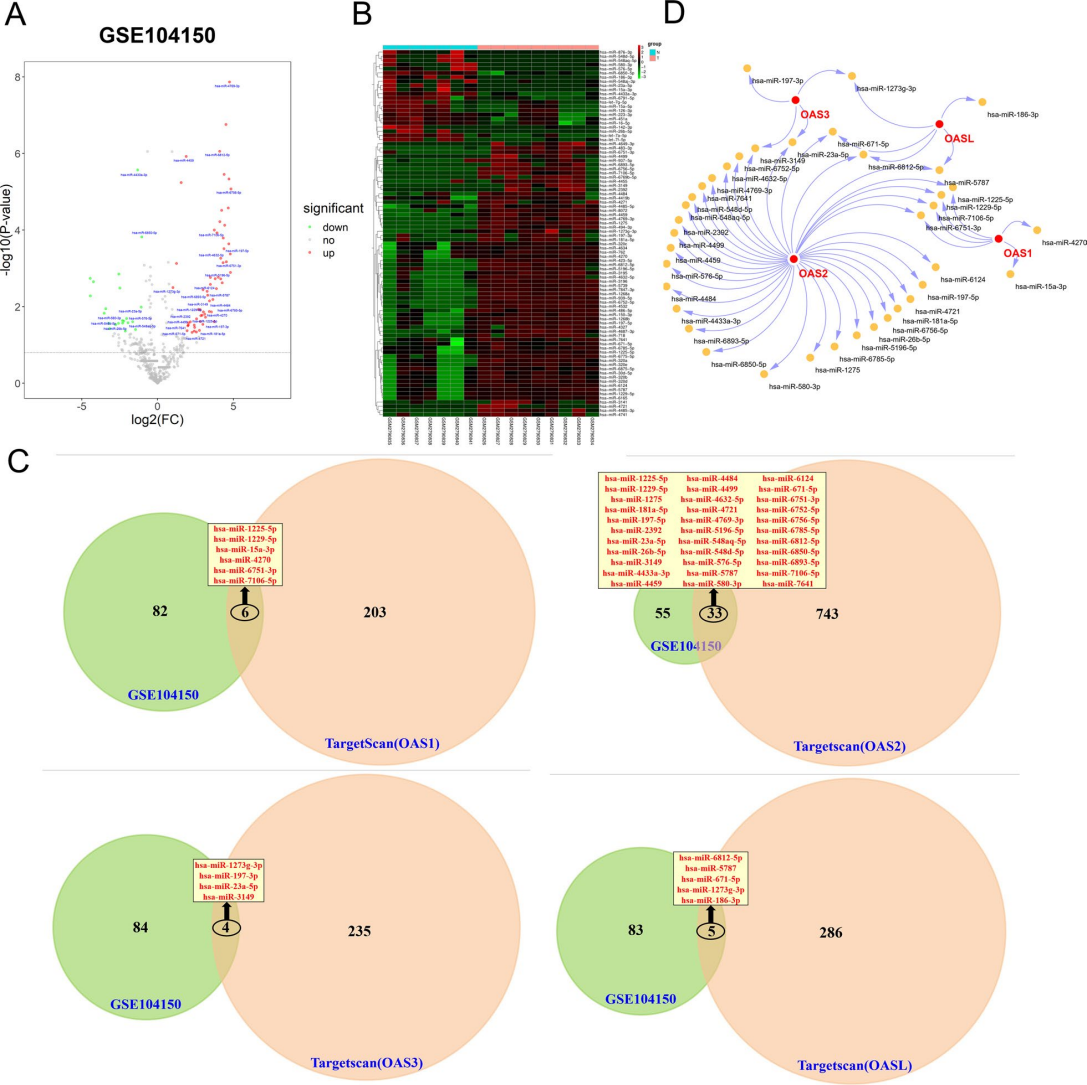
miRNAs that regulate OAS gene family. **A** Volcano plot of different expressed miRNAs in GSE104150. **B** Heatmap of differentially expressed miRNAs in GSE104150, with each column representing sample and each row indicating miRNAs. **C** Amount of miRNA from GSE104150 and Targetscan database, merged part is common miRNAs. **D** Common miRNAs derived from the intersection of GSE105150 and Targetscan database shown by cytoscape

To explore the regulatory mechanism of OAS gene family expression, we analyzed the miRNAs derived from GSE104150 (miRNA expression dataset of HF) and took intersection with the predicting results from Targetscan. As a result, total 88 different miRNAs were obtained from GSE104150, and among them, 66 miRNAs were upregulated and 22 miRNAs were downregulated (Fig. 8A, B; Additional file 1: Table S10). From the intersection of Targetscan and GSE104150, we found that 6, 33, 4, and 5 miRNAs regulated OAS1, OAS2, OAS3, and OASL, respectively, which were displayed in Fig. 8C, D.

Among these intersecting miRNAs, 10 miRNAs, including hsa-miR-15a-3p, hsa-miR-23a-5p, hsa-miR-26b-5p, hsa-miR-186-3p, hsa-miR-4433a-3p, hsa-miR-548aq-5p, hsa-miR-548d-5p, hsa-miR-576-5p, hsa-miR-580-3p, and hsa-miR-6850-5p, were down-expressed, this may be the reason for the high expression of OAS gene family in HF.

### Predicted chemicals and ingredients interacting with OAS gene family

**Fig. 9:**
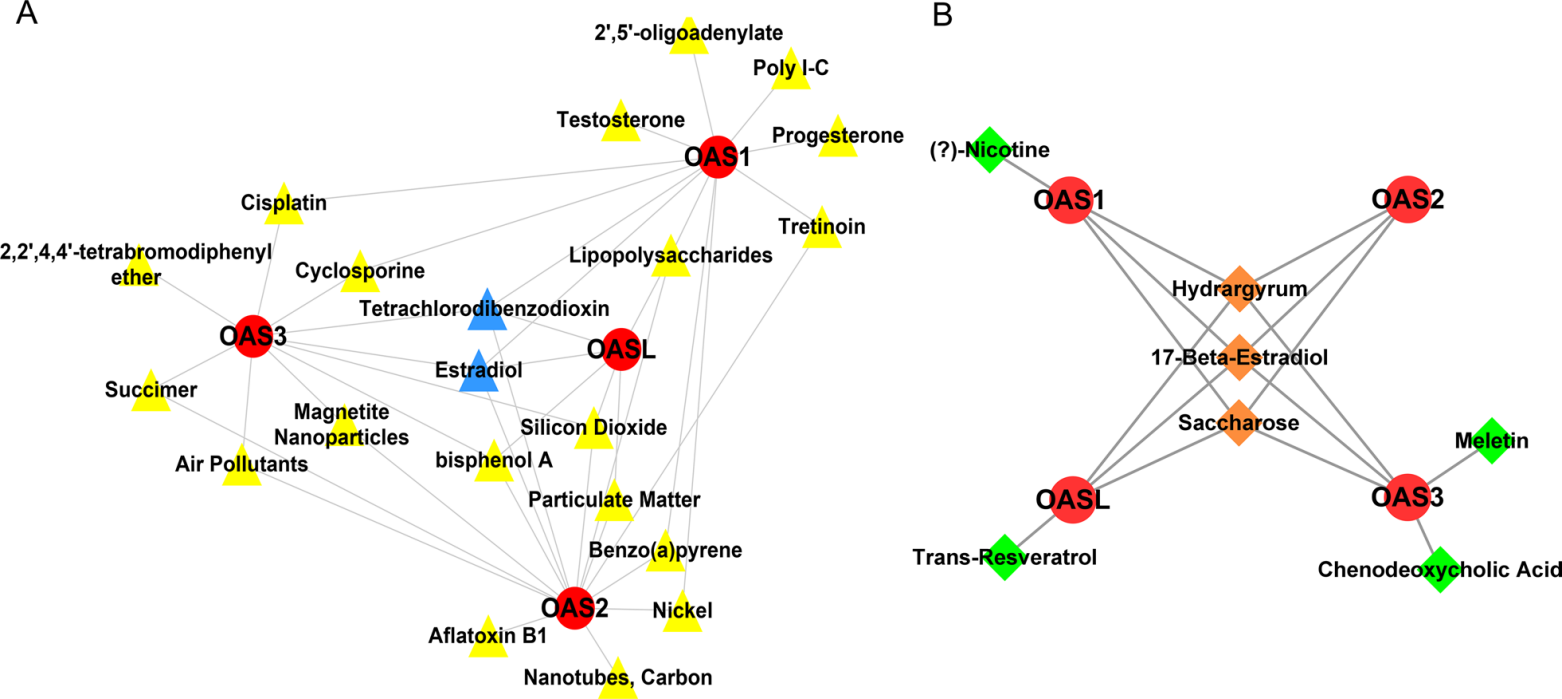
Chemical or ingredient that interacted with OAS gene family from CTD and SymMap database. **A** Chemicals interacted with OAS gene family. There are 12, 14, 10, and 6 chemicals interact with OAS1, OAS2, OAS3, and OASL, respectively. Red circle means OAS gene family, yellow triangle means chemical, blue triangle means common chemical. **B** Ingredients that reacted with OAS gene family. There are 4, 3, 5, and 4 ingredients acting on OAS1, OAS2, OAS3, and OASL, respectively. Red circles indicate OAS gene family, green diamonds represent drug, orange diamonds mean common ingredients

CTD and SymMap databases were used to predict the chemicals and ingredients that may interact with the OAS genes. Results of CTD analysis showed that 12, 14, 10, and 6 chemicals interacted with OAS1, OAS2, OAS3, and OASL, respectively. Notably, estradiol and tetrachlorodibenzodioxin were found to be the common chemicals regulating the four OAS genes (Fig. 9A). Results of SymMap analysis showed that 4, 3, 5, and 4 ingredients acted on OAS1, OAS2, OAS3, and OASL, respectively. Among them, 17-β-estradiol, hydrargyrum and saccharose were the common chemicals or ingredients that regulated the OAS gene family (Fig. 9B). The 17-β-estradiol, also named estradiol [37], were commonly recommended by CTD and SymMap databases.

### Results of docking analysis

**Fig. 10:**
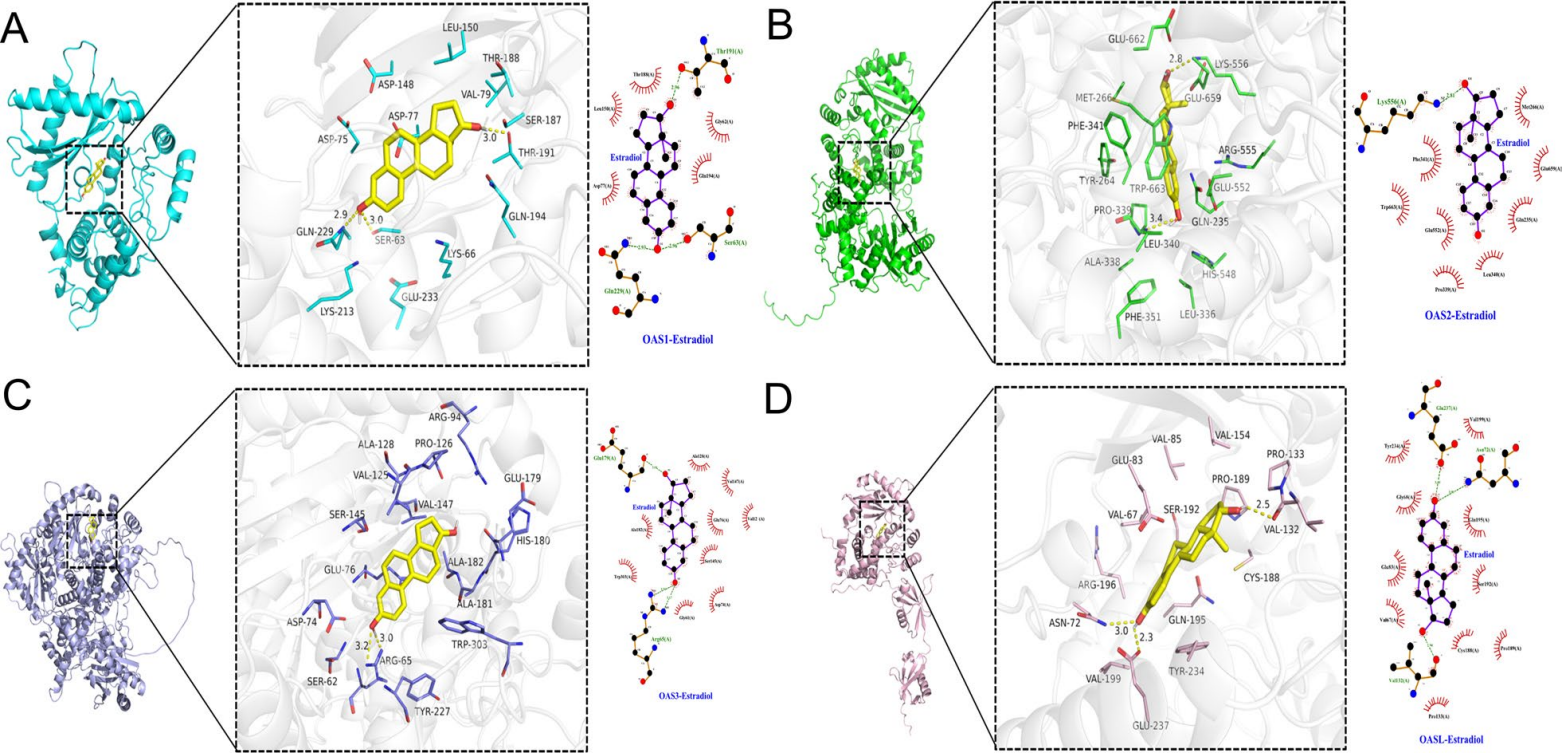
Docking analyses of affinity between OAS genes and chemicals/ingredients. **A** OAS1. **B** OAS2. **C** OAS3. **D** OASL

The 3D structure of OAS1 was obtained from PDB database (PDB ID: 4IG8), and the 3D structures of OAS2, OAS3, and OASL were downloaded from UniProt database. The binding energies of 17-β-estradiol with OAS1, OAS2, OAS3, and OASL were − 7.1 kcal·mol^−1^, − 7.6 kcal·mol^−1^, − 8.4 kcal·mol^−1^, and − 8.7 kcal·mol^−1^, respectively, suggesting that the binding between 17-β-estradiol and the four “receptors” (OAS proteins) were strong. From the results of ligand-receptor protein interaction, we found that 17-β-estradiol could form hydrophilic binding with SER63, GLN229, and THR19, and also had hydrophobic interactions with the amino acid residues ASP77, GLY62, GLN194, LEU150, and THR188 of OAS1 (Fig. 10A). In addition, 17-β-estradiol exhibited hydrophilic force with LEU340 and LYS556, while had hydrophobic force with PHE341, TRP663, GLU552, PRO339, LEU340, GLN235, GLU659, and MET266, of OAS2 (Fig. 10B). The 17-β-estradiol also had hydrophilic effects on the ARG65 of OAS3, while had hydrophobic effects on ALA182, TRP303, GLY61, ASP74, SER145, GLU76, VAL125, VAL147, and ALA128 of OAS3 (Fig. 10C). Furthermore, 17-β-estradiol showed hydrophilic binding with ASN72, GLU237 and VAL132, but exhibited hydrophobic interaction with TYR234, GLY68, GLU83, VAL67, CYS188, SER192, GLN185 and VAL199, of OASL (Fig. 10D). These forces made 17-β-estradiol stably binding to the pockets of the four OAS proteins.

## Discussion

The highlight of this study was the discovery of an important role of OAS gene family in the process of COVID-19 induced HF. HF is one of the major adverse consequences of COVID-19 [9]. The fact that OAS genes were highly expressed in both SARS-CoV-2-infected cardiomyocytes and human failing hearts provides us a reason to believe that some similar signaling molecules and/or pathways may mediate the developments of COVID-19 and HF, or in other words, there may be some similar or common molecular mechanisms in the two diseases, and OAS gene family may be the common genetic factors. Based on the present bioinformatic analysis (shown in Fig. 1 and Fig. 3) and previous reports, OAS genes are highly expressed in SARS-CoV-2 infected cardiomyocytes and the hearts of COVID-19 patients [23, 38–40], and our qPCR experiments further verified the high expressions of OAS genes in the myocardium of COVID-19-free HF cases (shown in Fig. 2 and Fig. 4). Previous studies have reported that OAS cluster variants are associated with greater risk of severe COVID-19, and OAS gene family plays an important role in the innate antiviral mechanisms linking to SARS-CoV-2 infection [40–42]. These findings may be associated with the immune dysregulation and cytokine storm leading to HF in COVID-19 patients [43, 44]. During the cytokine storm, a large number of inflammatory factors are produced, such as TNF-α, IL-1, IL-6, and IFN-γ, leading to severe inflammation, multi-organ failure and even death. Several previous studies have reported that cytokine storm can occur in COVID-19 patients, and the level of pro-inflammatory factors is positively correlated with disease severity [45, 46]. This may be a crucial reason for developing HF in COVID-19 cases.

To corelate OAS gene family with COVID-19 associated HF, it is worthy to mention the important roles of IFN and IFN-stimulated genes (ISGs) in the endogenous anti-virus processes. OAS gene family is closely related to the induction of IFN [21]. In COVID-19 patients, SARS-CoV-2 strongly triggers the expressions of many ISGs and activates immune cells. ISGs have immunopathogenic potentials, including overexpression of inflammatory genes. In some COVID-19 cases, the level of IFN-I is low at the early stage, leading to excessive viral proliferation; when the IFN-I reaches a high level at the advanced stage, it may be too late to be rescued. Massive viral replication and early immune escape result in hyperactivation of pro-inflammatory responses [47–49]. In severe COVID-19 cases, IFN-I response arouses an excessive inflammatory response by promoting TNF/IL-1β-driven inflammation leading to cytokine storm [50]. As the critical members of innate immunity, OAS genes play important roles in immune responses and even the cytokine storm, this may be one reason why COVID-19 can develop to HF. Zhang et al. [51] found a highly preserved transcriptional profile of IFN-I genes for COVID-19 complementary diagnosis, and OAS genes were included in the profile. These evidences suggest the important role of OAS gene family in COVID-19. We noticed that IFN-I related genes, such as MX1, RSAD2, and IFIT2, were listed in the OAS-correlated-genes (shown in Additional file 1: Table S9). Yu et al. also reported that MX1, RSAD2 and IFIT3 (belonging to another gene family of IFIT2) are hub genes in HF. These findings suggest a crucial role of IFN-I related genes in HF [52]. Adeghate et al. [53] reported that SARS-CoV-2 invasion can damage the heart via the following mechanisms: (1) infiltration of inflammatory cells into the myocardium; (2) cardiomyocyte death caused by pro-inflammatory cytokines; (3) virus-induced damage of endothelial cells and micro-thrombosis; (4) hypoxia caused by respiratory failure. Among these mechanisms, OAS genes may play roles at least in some of them, for example, the immune responses.

COVID-19 is associated with many inflammation-related signaling pathways, such as interleukin-6/Janus kinase/STAT (IL-6/JAK/STAT) pathway, interferon (IFN) cell signaling pathway, tumor necrosis factor-α/nuclear factor-kappa (TNFα/NF-κB) pathway, toll-like receptor (TLR) pathway, T-cell receptor (TCR) pathway, etc. [54–56]. Our study on COVID-19 dataset reveals that inflammation-related pathways, such as hsa04668 (TNF signaling pathway), hsa04210 (Apoptosis), and hsa04630 (JAK-STAT signaling pathway), are consistent with the above viewpoint. These signaling pathways also have important biological functions in HF [57–59]. In addition, our results show that after cardiomyocytes being infected by SARS-CoV-2, some HF-associated biological pathways became prevalent, such as hsa04260 (cardiac muscle contraction) and hsa05415 (diabetic cardiomyopathy), which directly indicates the signaling by which COVID-19 causes HF. By analyzing the DEGs in HF dataset GSE120852, we found that some signaling pathways associated with the DEGs, such as GO: 0002252 (BP: immune effector process), GO:0002683 (BP: negative regulation of immune system process), GO:0050727 (BP: regulation of inflammatory response), hsa05171 (coronavirus disease-COVID-19), hsa04210 (apoptosis), and hsa04064 (NF-kappa B signaling pathway), are COVID-19 or inflammation related pathways. Especially, the term hsa05171 (coronavirus disease-COVID-19) from the HF dataset fully illustrates the close relationship of COVID-19 with HF.

Currently, there is no specific and effective drug for COVID-19. Thus, development of this kind of drugs is in urgent need. Some antiviral drugs have the potential to treat COVID-19, such as remdesivir, lopinavir/ritonavir, and interferon β-1a [60]. In the present study, we predicted some potential chemicals or ingredients which regulate the expression of OAS genes by analyzing the CTD and SymMap databases (shown in Fig. 9). Notably, we found that estradiol is one of them. Except for regulating the sexual system, estradiol plays an important role in anti-inflammation and suppression of virus-induced innate immune inflammatory response. High physiological concentration of estradiol can reduce the production of pro-inflammatory cytokines, such as IL-6, IL-1β, TNF-α, and CCL2, and prevent migration of monocytes and neutrophils into inflamed tissues. Therefore, under the influence of estradiol, immune dysregulation caused by cytokine storm in COVID-19 is ameliorated [61, 62]. Women generally show better immune responses to viruses than men, men are more susceptible to severe COVID-19 [63, 64]. A study proposed that a combination of estradiol with vitamin D and quercetin can be used to relieve COVID-19 [65]. Estradiol also has a certain protective effect on cardiovascular diseases [66, 67]. Estradiol can rescue severe HF through the classical estrogen receptor beta (ERβ), which is present in the heart [68]. Frump et al. [69] reported that estradiol can protect the function of right ventricle in pulmonary hypertension via BMPR2 and apelin. These evidences suggest the protective effect of estradiol on the heart, including the damaged heart in COVID-19.

The study has some limitations. We were unable to obtain cardiac tissues from COVID-19 and HF patients to verify the expression and mechanism of OAS gene family in these diseases. The effect of estradiol on OAS gene family needs further experimental verification. These issues warrant future studies.

## Conclusions

**Fig. 11:**
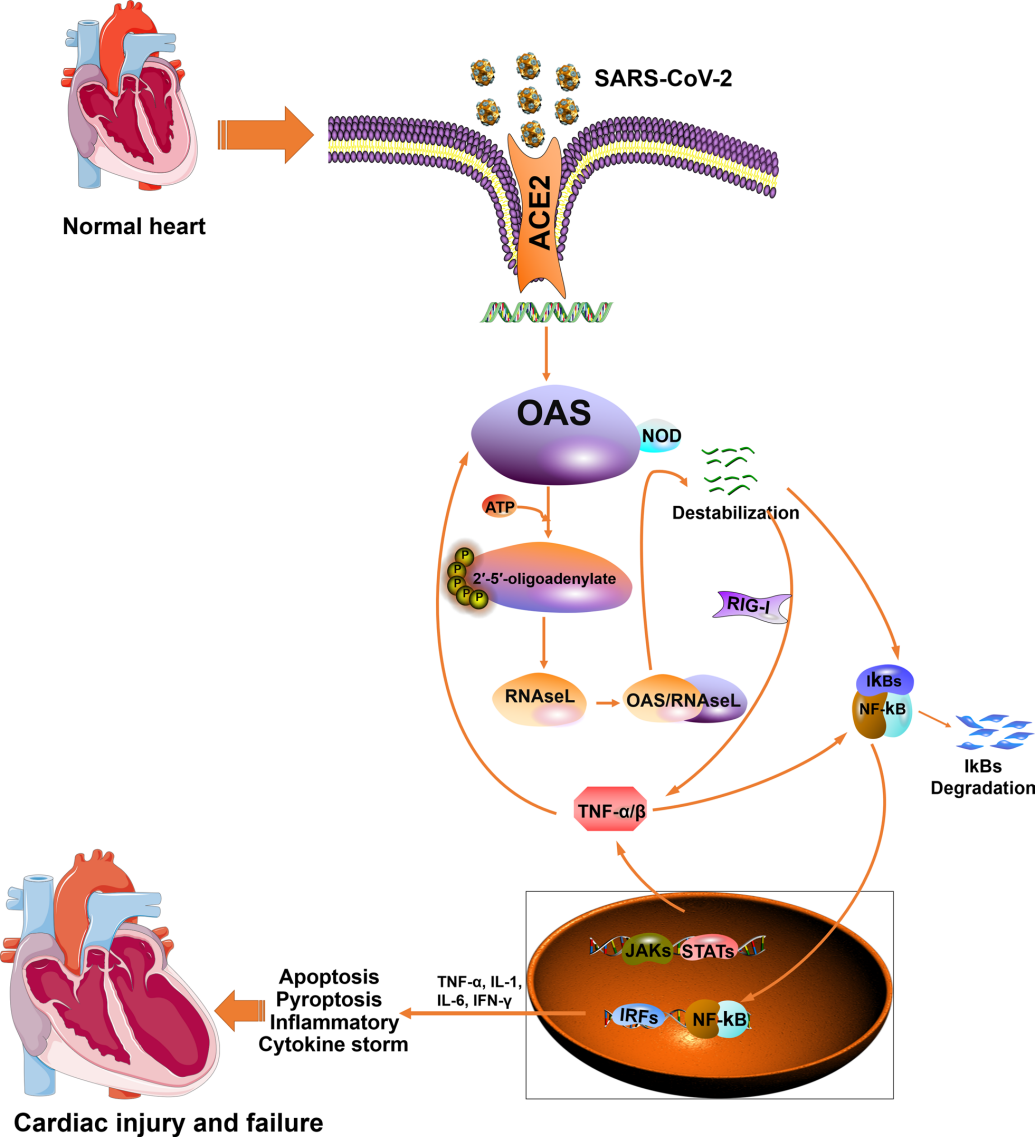
Scheme showing the putative signaling by which OAS genes affect the heart and induce HF after myocardial infection by SARS-CoV-2

OAS genes are highly expressed in SARS-CoV-2 infected cardiomyocytes, cardiac tissues of COVID-19 patients, and COVID-19-free human failing hearts, and are potentially strong linkers of COVID-19 with HF. OAS genes interact with the invaded SARS-CoV-2 and activates the OAS/RNaseL antiviral system to degrade the viral RNAs. The degraded small viral RNA fragments activate NF-kB inflammation-related pathways and produce inflammatory factors or even cytokine storms, ultimately leading to cell death and HF. A detailed flow chart is shown in Fig. 11.

## Supplementary Information

**Additional file 1: Table S1**. Primer Sequences for qPCR. **Table S4**. GO analysis of 1448 DEGs in GSE150392. **Table S5**. KEGG pathway analysis of 1448 DEGs in GSE150392. **Table S6**. GO analysis of 239 common DEGs in GSE120852. **Table S7**. KEGG pathway analysis of 239 common DEGs in GSE120852. **Table S8**. Different expression miRNAs from GSE104150. **Table S9**. Intersecting miRNAs explored from GSE104150 and Targetscan.

**Additional file 2.** **Table S2.** DEGs from GSE150392.

**Additional file 3.** **Table S3.** DEGs (diff1, diff2, diff3, and diff4) from GSE120852.

## Data Availability

All data in this study are available from the corresponding author upon reasonable request. All authors read and approved the final manuscript. The datasets (GSE150392, GSE120852, GSE147507, GSE 179850 and GSE104150) presented in this study can be found in NCBI Gene Expression Omnibus.

## Abbreviations

HF: Heart failure
COVID-19: Coronavirus disease 2019
SARS-CoV-2: Severe acute respiratory syndrome coronavirus 2
OAS: 2ʹ,5ʹ-Oligoadenylate synthetase
DEGs: Differentially expression genes
miRNA: MicroRNAs
RIG-1: Acid-inducible gene-I
IRF: IFN regulatory factor
NCBI-GEO: National center of biotechnology information- gene expression omnibus
NF: Non-failing
LV: Left ventricles
RV: Right ventricles
Bi-HF: Biventricular failure
NHBE: Human lung epithelial
GO: Gene ontology
KEGG: Kyoto encyclopedia of genes and genomes
BP: Biological processes
CC: Cellular components
MF: Molecular functions
SPF: Specific-pathogen-free
TAC: Transverse aortic constriction
HW/BW: Heart weight/body weight
EF: Ejection fraction
FS: Fractional shortening
LVPWd: Left ventricular posterior wall thickness at end diastole
LVPWs: Left ventricular posterior wall thickness at systole
Ang II: Angiotensin II
qPCR: Quantitative real-time PCR
PPI: Protein–protein interaction
DMNC: Density of maximum neighborhood component
CTD: Comparative toxicogenomics database
TCM: Traditional Chinese Medicine
MM: Modern medicine
ISGs: IFN-stimulated genes
