# Supplementary material for: Role of OAS gene family in COVID-19 induced heart failure
Source: J Transl Med. 2023 Mar 22;21:212. doi: 10.1186/s12967-023-04058-x (PMC10031198; doi:10.1186/s12967-023-04058-x)
Supplement: Supplementary file 1 — Additional file 1: Table S1. Primer Sequences for qPCR. Table S4. GO analysis of 1448 DEGs in GSE150392. Table S5. KEGG pathway analysis of 1448 DEGs in GSE150392. Table S6. GO analysis of 239 common DEGs in GSE120852. Table S7. KEGG pathway analysis of 239 common DEGs in GSE120852. Table S8. Different expression miRNAs from GSE104150. Table S9. Intersecting miRNAs explored from GSE104150 and Targetscan. [file 12967_2023_4058_MOESM1_ESM.docx]

**Role of OAS gene family in COVID-19 induced heart failure**

Li-Juan Gao^*‡^, Zhong-Mei He^‡^, Yi-Ying Li, Rui-Rui Yang, Min Yan, Xuan Shang, Ji-Min Cao^*^

From the Key Laboratory of Cellular Physiology at Shanxi Medical University, Ministry of Education, and the Department of Physiology, Shanxi Medical University, Taiyuan, China

^‡^ These authors contributed equally to this work.

^*^ Correspondence:

Ji-Min Cao, MD, PhD. Department of Physiology, Shanxi Medical University, Taiyuan, Shanxi 030001, P. R. China. Email: [caojimin@sxmu.edu.cn](mailto:caojimin@sxmu.edu.cn). ORCID: 0000-0002-6546-555X

Li-Juan Gao. MS. Department of Physiology, Shanxi Medical University, Taiyuan, Shanxi 030001, P. R. China. Email: gaolijuan@sxmu.edu.cn. ORCID: 0000-0002-2274-8445

| **Table S1.**  **Primer Sequences for qPCR** | | | |
| --- | --- | --- | --- |
| **Genes from Rattus norvegicus (rat)** | **Forward (F) and Reverse (R) Primer Sequence (5’ – 3’)** | **Genes of Mus musculus (mmu)** | **Forward (F) and Reverse (R) Primer Sequence (5’ – 3’)** |
| **rat-GAPDH-F** | AGTGCCAGCCTCGTCTCATA | **mmu-GAPDH-F** | AGGTCGGTGTGAACGGATTTG |
| **rat-GAPDH-R** | GATGGTGATGGGTTTCCCGT | **mmu-GAPDH-R** | GGGGTCGTTGATGGCAACA |
| **rat-ANP-F** | CTTCTCCATCACCAAGGGCTTCTTC | **mmu-ANP-F** | TCTTCCTCGTCTTGGCCTTT |
| **rat-ANP-R** | TCCAGGTGGTCTAGCAGGTTCTTG | **mmu-ANP-R** | CCAGGTGGTCTAGCAGGTTC |
| **rat-BNP-F** | CCAGTCTCCAGAACAATCCACGATG | **mmu-BNP-F** | TGGGAGGTCACTCCTATCCT |
| **rat-BNP-R** | GCCTTGGTCCTTTGAGAGCTGTC | **mmu-BNP-R** | GGCCATTTCCTCCGACTTT |
| **rat-β-Mhc-F** | GCCCCAAATGCAGCCAT | **mmu-β-MHC-F** | ACTGTCAACACTAAGAGGGTCA |
| **rat-β-Mhc-R** | CGCTCAGTCATGGCGGAT | **mmu-β-MHC-F** | TTGGATGATTTGATCTTCCAGGG |
| **rat-OAS1-F** | GGAAGTGCCGGTGGATGAG | **mmu-OAS1-F** | TGGAGCAGGATCTGAGGAGCATC |
| **rat-OAS1-R** | CCCTCACTGTGTCAAGGTCC | **mmu-OAS1-R** | GAGCACACAGGGCATCTATGACTTC |
| **rat-OAS2-F** | CTCACCTCCGCCATTGCTATATGC | **mmu-OAS2-F** | GAGTTCTCAGACTGCTTCACCACAC |
| **rat-OAS2-R** | CAGTCCTGATACCTTGCGTGATGTC | **mmu-OAS2-R** | CACTTCTCCTGGCACTGTTCATACC |
| **rat-OAS3-F** | CGTGTCAGCAGAAGCAGAGGTTC | **mmu-OAS3-F** | GTCAAAGGCGTCCAGGATCTTACAG |
| **rat-OAS3-R** | GCCTAGCAGCGTCTTGGATGTC | **mmu-OAS3-R** | GCAGGCACCAGGCTAACATCTATC |
| **rat-OASL-F** | CGCCGAGGTCTATGTGAATCTGATC | **mmu-OASL-F** | CATCATTGTCCTTACCCACA |
| **rat-OASL-R** | TGACCAACCGTAGGAGGCTCTTC | **mmu-OASL-R** | TAGGTCCCAAAGCATCGT |

**Table S2.** DEGs from GSE150392

Due to the large content of table S2, it is listed as “Additional file 2” in an excel.

**Table S3.** DEGs (Diff1, diff2, diff3, and diff4) from GSE120852

Due to the large content of table S3, it is listed as “Additional file 3” in an excel.

| **Table S4. GO analysis of 1448 DEGs in GSE150392** | | | | |
| --- | --- | --- | --- | --- |
| **Category** | **Term** | **Description** | **LogP** | **Symbols** |
| BP | GO:0071345 | cellular response to cytokine stimulus | -20.64 | BIRC3,KLF5,CD44,CCR7,CNTF,CSF1,CSF2RB,CYP1B1,DUSP1,EGR1,FABP4,GATA3,GBP3,GPD1,CXCL1,CXCL2,CXCL3,HAS2,HES1,ICAM1,IRF8,IFIT2,IFIT3,IL1B,IL1R1,IL6,IL7R,CXCL8,CXCR1,IL12RB2,IL15RA,IRAK2,IRF1,IRF7,ITGA4,LGALS9,LOX,MRC1,MT3,MX1,NFKB1,NFKBIA,OAS1,OAS2,OSM,PCK1,PIGA,MAPK13,PTGIS,CCL2,CCL5,CCL7,CCL20,CXCL6,SELPLG,SP100,SSTR1,THBS1,TLR4,TNFRSF1B,TRAF1,UGCG,VEGFC,TRIM25,CXCR4,IFITM1,OASL,IL1RL1,OSMR,CRLF1,KLF4,NR1D1,GFPT2,SH2B3,CCL26,CDC42EP2,IL24,SBNO2,CLCF1,ANKRD1,IL20RA,CACTIN,SOX17,RBM15,ZC3H12A,IL33,GBP5,IFNL1,AGTR1,BCL6,BMP6,BMPR1B,C3AR1,CD14,CD36,CD68,ADGRE5,EPHA2,ELF3,F2RL1,FOS,FPR3,HMGB2,HPR,HRH1,ITGAM,JUN,LY75,PIK3CD,PTAFR,PTGER4,PTGFR,PTGS1,PTGS2,PTX3,RELB,SAA1,SELE,TFR2,TNFAIP3,TNFSF4,CSRP3,AOC3,SPHK1,IL17B,GAL,TREM1,ODAM,NFKBIZ,APOL3,LOXL3,NFKBID,C2CD4A,CYP1A1,GCH1,NR4A1,HPGD,IL12A,MAOB,SERPINE1,NOCT,NOD2,PDCD1LG2,CMPK2,BCL3,HLA-E,OAS3,UPK1B,H2BC8,H2BC7,H2BC6,ISG15,TRDN,IFI44,BAIAP2L1,DHX58,LYG1,DDIT3,ADCY5,KLF9,INHBA,PDK4,RXRG,SGK1,STC1,WNT7B,NR4A3,HAND2,ERRFI1,TESC,BRINP2 |
| BP | GO:0003013 | circulatory system process | -16.52 | ACTC1,AGTR1,ATP2A3,BMP6,C3AR1,CASQ2,CAV3,CD36,DRD1,EMP2,F2RL1,FGF12,GCH1,GJA5,GUCY1A1,HRH1,HRH2,HSD11B2,HTR7,MYL2,MYL4,NPPA,ABCB1,PTAFR,PTGS1,PTGS2,SCN2B,SGK1,SLC2A3,SLC2A5,SLC5A1,SLC5A3,SLC5A5,SLC8A1,SLC15A2,SLC22A1,SLC22A3,TNNC1,TNNI1,TNNI3,TNNT2,VEGFC,CXCR4,CSRP3,SLC7A5,TCAP,APLN,PER2,GPR37L1,HCN4,TRDN,CORIN,SLC38A3,EMILIN1,SLC38A2,SUCNR1,TBX20,MYL7,ACE2,ADM2,SPX,GRIP2,SLC38A5,C2CD4A,ADM5,CCR7,CRABP2,IL7R,L1CAM,MT3,TMOD1,WNT7B,CDK5R1,KIAA0319,USH1C,CCL26,CDC42EP2,TMSB15A,LPAR3,PPP1R15A,TRPV2,BAIAP2L1,BAIAP2L2,SLC12A8,LMOD2 |
| MF | GO:0048018 | receptor ligand activity | -16.29 | ADCYAP1,ASIP,BMP6,CNTF,CSF1,CTF1,DPP4,TYMP,FGF7,FGF12,GDF9,CXCL1,CXCL2,CXCL3,HMGB2,IL1B,IL6,CXCL8,IL11,IL12A,INHBA,LGALS3,LIF,NPPA,NTF4,OSM,PYY,RLN2,CCL2,CCL5,CCL7,CCL20,CXCL6,SECTM1,SFRP2,STC1,TG,LEFTY2,TRH,TNFSF4,VEGFC,WNT7B,STC2,TNFSF9,APLN,CRLF1,GDF15,CXCL14,CCL26,CDC42EP2,CSPG5,NRG3,IL24,CLCF1,GREM1,IL17B,GAL,ADM2,SPX,INHBE,IL33,DAND5,IFNL1,TAFA2,DKKL1,DKK4,LYPD6,PCSK9,GATA3,ITGA5,TRAF1,OSMR,SH2B3,NES,SPRED1 |
| CC | GO:0030017 | sarcomere | -15.15 | ACTA1,ACTC1,CASQ1,CASQ2,CAV3,CRYAB,DES,HRC,HSPB1,MYL2,MYL4,SLC8A1,TMOD1,TNNC1,TNNI1,TNNI3,TNNT2,CSRP3,TCAP,SQSTM1,MYL9,NEBL,LDB3,SMPX,ANKRD2,ITGB1BP2,ANKRD1,ASB2,TRIM54,MYL7,SYNPO2L,ADPRHL1,C10orf71,KLHL40,FBXL22,KY,LRRC10,LMOD2,MYZAP,CDK5R1,ATF3,KLF5,CDK1,EGR1,EGR2,FOS,GJA5,HLF,NR4A1,FOXN2,NPPA,PDGFRA,RXRG,HAND1,HCN4,MAFF,SRPK3,SIX4,TBX20,HOPX,SMYD1,SORBS1,ANLN,FRMD6,PHACTR1,FRMD3,HES1,LOX,NFATC2,CRIP1,FGF12,FOXL1,GATA3,HAS2,JUN,MB,SFRP2,SNAI1,TNFRSF1B,APLN,HAND2,ADAMTS1,EMILIN1,HSPB7,SOX17,RBM15,DNAAF4,SPRED1,DAND5,BIRC5,CDC20,GRB7,NEK2,RPL3L,PRC1,CCNB2,AURKB,DLGAP5,NDC80,PLK4,NOCT,CDCA8,CENPH,FITM1,ZPBP2,BMPR1B,CCKAR,EPHA2,EPHB1,ITGA4,L1CAM,NPTX1,NTF4,NR4A2,NECTIN1,SGK1,TPBG,WNT7B,CSPG5,NES,ZNF335,VSTM2L,ACTBL2 |
| BP | GO:0044057 | regulation of system process | -13.66 | PARP1,AGTR1,ATP2A3,BMP6,CASQ1,CASQ2,CAV3,CRY1,DES,TYMP,EGR2,EMP2,F2RL1,FGF12,GCH1,GDF9,GJA5,GUCY1A1,NR4A1,HRH1,HRC,HRH2,INHBA,MYL2,MYL4,NPPA,NPTX1,NPTX2,FXYD1,PTAFR,PTGS2,RGS4,SCN2B,SGCA,SGK1,SLC8A1,STC1,TNFRSF1B,TNNC1,TNNI1,TNNI3,TNNT2,NR4A3,CSRP3,APLN,PER2,SPHK1,HAND2,HCN4,MYL9,CORIN,NMU,CACNG4,HSPB7,KCNIP2,GAL,ERRFI1,ACE2,WNK4,ADM2,ZC3H12A,SPX,HOPX,MLIP,C1QTNF1,ADM5 |
| BP | GO:0030155 | regulation of cell adhesion | -13.39 | BCL6,BMP6,CD36,CD44,CDH1,CCR7,MAP3K8,CSF1,CYP1B1,DPP4,DUSP1,EPHA2,EMP2,FUT1,GATA3,HAS2,HFE,HLA-E,HES1,TNC,IL1B,IL6,IL7R,CXCL8,IL12A,IRF1,ITGA4,ITGA5,LGALS3,LGALS9,LIF,TACSTD2,MYO10,NEDD9,SERPINE1,PCDH8,PCK1,SERPINE2,PLAUR,PLG,PTAFR,SAA1,CCL2,CCL5,SELE,SFRP2,TGFBI,THBS1,THY1,TNFSF4,CXCR4,NR4A3,TNFSF9,CD83,KLF4,SH2B3,CSPG5,SPINK5,EMILIN1,EGFL6,GREM1,TBX21,TESC,FERMT1,CASS4,NOD2,NFKBIZ,ZC3H12A,PDCD1LG2,FERMT3,CARD11,LOXL3,NFKBID,UBASH3B,C1QTNF1,C2CD4A,IL4I1,IFNL1,VWC2,GPR183,F2RL1,IL15RA,INHBA,ITGAM,JUNB,NFATC2,PDGFRA,PLA2G4A,TLR4,TNFAIP3,TNFRSF1B,SPHK1,IL1RL1,NR1D1,TSPAN32,CLCF1,GAL,SH3RF1,ZNF335,IL33,FOS,HMGB2,HOXA5,IRF7,LOX,NFKBIA,KLF10,BRPF1,H4C3,H4C2,ISG15,RBM15,MTURN,BST2,CSF2RB,HHEX,CFB,C3AR1,FPR3,IL1R1,MICB,MOG,PIK3CD,BTN3A1,SUSD4,PARP9,RSAD2,GBP5 |
| BP | GO:0001819 | positive regulation of cytokine production | -13.24 | ADCYAP1,BCL3,C3AR1,CD14,CD36,CCR7,CYP1B1,DDIT3,EGR1,F2RL1,GATA3,HLA-E,HMGB2,HSPB1,IRF8,IL1B,IL1R1,IL6,IL12A,IL12RB2,IRF1,IRF7,LGALS9,OAS1,OAS2,OAS3,OSM,SERPINE1,PIK3CD,MAPK13,PTAFR,PTGER4,PTGS2,SAA1,THBS1,TLR4,TNFSF4,NR4A3,SLC7A5,SPHK1,IL1RL1,CD83,ISG15,BTN3A1,SULF1,DDX58,IL17B,TBX21,FERMT1,NOD2,IFIH1,DHX58,CARD11,IL33,RSAD2,GBP5,MCOLN2,IFNL1,AGTR1,BIRC3,BCL6,CYP19A1,FABP4,TNC,MICB,MMP9,NFKB1,NFKBIA,NT5E,PTGIS,CCL5,CXCL6,SELE,TNFAIP3,TNFRSF1B,OSMR,KLF4,PTGES,NR1D1,TSPAN32,TRIM38,SPINK5,EMILIN1,USP18,SBNO2,SAMHD1,PARP14,SUSD4,PBK,SUCNR1,CACTIN,ACE2,NFKBIZ,ZC3H12A,PARP9,C2CD4A,DTX3L,NCF1,BMP6,CNTF,CSF1,CXCL8,PLG,CCL7,TPBG,VEGFC,CXCR4,OASL,CCL26 |
| BP | GO:0009615 | response to virus | -12.17 | BCL3,BST2,CYP1A1,F2RL1,IFI6,GATA3,GBP3,HSPB1,IFIT2,IFIT3,IL6,IL12A,IRF1,IRF7,ISG20,LGALS9,MX1,MX2,NFKB1,OAS1,OAS2,OAS3,PMAIP1,CCL5,TNFSF4,TRIM25,ZNF175,CXCR4,FOSL1,IFITM1,OASL,ISG15,TRIM22,IFI44,IFI44L,DDX58,SAMHD1,TBX21,IFIH1,DHX58,ZC3H12A,PARP9,RSAD2,SLFN11,DTX3L,MLKL,IFNL1,CXCL8,PTX3,TOP2A,CH25H,TRIM14,TRIM38,PARP10,SP100 |
| BP | GO:0010817 | regulation of hormone levels | -11.56 | ADCY5,ADCYAP1,ADH1B,AGTR1,ALDH1A3,BMP6,CCKAR,CRABP2,CRY1,CRYM,CTSK,CYP1A1,CYP1B1,CYP19A1,DPP4,EGR1,GATA3,GDF9,GPR27,HADH,HFE,ICA1,IL1B,IL6,IL11,INHBA,LIF,NFKB1,OSM,PCSK2,PDGFRA,PTPRN,RPE65,CCL5,SLC5A5,TFR2,TG,TPO,TRH,VSNL1,HSD17B8,SLC7A5,STC2,PER2,NR1D1,CORIN,GAL,ACE2,CHST8,ALDH8A1,DHRS11,LRP5L,C1QTNF1,ACVR1C,PLB1,IL4I1,ACHE,CAMK2A,DRD1,F2RL1,IL12A,ITGAM,LGALS9,MAOB,OAS2,PLA2G4A,PTAFR,RAB27B,SAA1,SGK1,STC1,TLR4,TNFRSF1B,VEGFC,HAP1,PTGES,CSPG5,SEC24A,ANKRD1,MCTP2,WNK4,SPX,RSAD2,SYT2,STXBP5 |
| BP | GO:0044706 | multi-multicellular organism process | -11.50 | ADCY7,ADCYAP1,CYP1A1,EMP2,FOS,FOSB,HPGD,HSD11B2,IL1B,ITGA5,JUNB,LGALS9,LIF,MMP9,NPPA,OVGP1,PAPPA,SERPINE2,PTAFR,PTGFR,PTGS2,RLN2,STC1,FOSL1,STC2,CORIN,SLC38A3,MAFF,DKKL1,CLIC5,SLC38A2,ACE2,RXFP1 |
| BP | GO:0043408 | regulation of MAPK cascade | -11.27 | ADCYAP1,ATF3,CALCR,CAV3,CD36,CD44,CCR7,DUSP1,DUSP4,DUSP6,GPR183,EPHA2,EPHB1,F2RL1,FGFR3,FOXM1,ICAM1,IL1B,IL6,IL11,JUN,LGALS9,LIF,MT3,GADD45B,NPPA,OSM,PDGFRA,PER1,CCL2,CCL5,CCL7,CCL20,SFRP2,THBS1,TLR4,TPBG,TRAF1,WNT7B,BCAR3,SPHK1,GPR37L1,HAND2,GDF15,RAPGEF2,SH2B3,CCL26,NPFFR2,EMILIN1,LPAR3,ERRFI1,ULK4,DOK5,PBK,SH3RF1,ACE2,NOD2,WNK2,ZC3H12A,DUSP16,CAVIN3,C1QTNF1,SH3RF2,SPRED1,MTURN,EMC10,SPRED3,NCF1,NEDD9,RASGRF2,RGS4,RGS16,THY1,RGN,TBC1D2B,ODAM,TBC1D2,GRHL3,AGTR1,ATP2A3,HMGB2,PLCB2,PMAIP1,PTAFR,SELE,PPP1R15A,ACVR1C,PLIN5 |
| BP | GO:0006936 | muscle contraction | -11.16 | ACTA1,CASQ2,CAV3,CKMT2,CRYAB,DES,DRD1,FGF12,HRC,HTR7,MYL2,MYL4,PGAM2,FXYD1,SCN2B,SGCA,SLC8A1,TMOD1,TNNC1,TNNI1,TNNI3,TNNT2,CXCR4,CSRP3,TCAP,HCN4,TRDN,SULF1,SMPX,ANKRD2,KCNIP2,TBX20,GRIP2,RCSD1,LMOD2,GUCY1A1,IL1B,NPPA,ACTC1,MYL7,GATA3,GJA5,HAS2,HES1,JUN,SFRP2,SNAI1,HAND1,HAND2,ADAMTS1,ANKRD1,ASB2,SOX17,RBM15,SYNPO2L,ADPRHL1,CNTF,LIF,DAND5,HOPX,ADCY5,ALDH1A3,GCH1,USH1C,PCDH15 |
| BP | GO:0043269 | regulation of ion transport | -11.04 | CALCR,CAMK2A,CASQ1,CASQ2,CAV3,CTSS,DRD1,FGF12,HFE,HRC,HES1,IL1B,KCND2,KCNG1,KCNJ6,KCNQ2,KCNS1,LGALS3,MAOB,MMP9,NPPA,PDZK1,PER1,ATP8B1,ABCB1,SERPINE2,PLA2G4A,FXYD1,PTAFR,PTGS2,RASGRF2,RCVRN,RGS4,SCN2B,CCL2,CCL5,SLC8A1,STC1,THY1,TRH,CXCR4,KCNAB1,STC2,PER2,HAP1,RGN,PTGES,HCN4,TRDN,SLC38A3,CACNG4,KCNIP2,GAL,TRPV2,CLIC5,TESC,DPP10,ACE2,WNK4,WNK2,UBASH3B,SYT2,NKAIN4,KCNG3,DHRS7C,PCSK9,NALCN,ANO9,SGK1,SLC5A3,SGK2,THBS1,NR4A3,SORBS1 |
| BP | GO:0061061 | muscle structure development | -11.03 | ACTA1,ACTC1,ATF3,KLF5,CASQ1,CAV3,CDK1,CHD2,CNTF,CRYAB,CTF1,DES,EGR1,EGR2,FOS,HLF,NR4A1,HES1,FOXN2,IFRD1,LIF,LOX,MYL2,NFATC2,PDGFRA,PLG,RXRG,SGCA,SLC8A1,TMOD1,TNNC1,TNNI1,TNNI3,TNNT2,CSRP3,TCAP,HAND1,MYL9,NEBL,LDB3,MAFF,ANKRD2,ITGB1BP2,SRPK3,ANKRD1,ASB2,SIX4,TBX20,SYNPO2L,ADPRHL1,KLHL40,SMYD1,LRRC10,LMOD2 |
| BP | GO:0007167 | enzyme-linked receptor protein signaling pathway | -10.49 | PARP1,BMP6,BMPR1B,CSF1,CTF1,EGR1,EPHA2,EPHB1,FGF7,FGF12,FGFR3,FOS,GALNT3,GDF9,GRB7,GUCY2F,HFE,HIVEP1,HPGD,HSPB1,INHBA,JUN,LIF,LOX,MMP9,NEDD9,NPPA,NTF4,PDGFRA,PDK4,PIK3CD,RPE65,SKIL,LEFTY2,VEGFC,NR4A3,CSRP3,BCAR3,CDK5R1,HAP1,DOK2,OSMR,GDF15,RAPGEF2,MVP,SH2B3,SORBS1,NRG3,SULF1,GREM1,SLC2A8,DOK5,CASS4,SMURF1,ARHGEF28,CSRNP1,SHCBP1,INHBE,UBASH3B,ACVR1C,FAM83G,CD44,DDIT3,DUSP6,GATA3,HAS1,HAS2,NR4A1,HES1,CXCL8,CCL2,CCL5,SPINT1,THBS1,TLR4,TNFRSF1B,SPHK1,KLF4,ANKRD1,ERRFI1 |
| BP | GO:0050900 | leukocyte migration | -9.76 | CCR7,GPR183,F2RL1,GATA3,CXCL1,CXCL2,CXCL3,HRH1,ICAM1,IL1B,IL6,CXCL8,CXCR1,ITGA4,LGALS3,MMP9,PIK3CD,PLG,SAA1,CCL2,CCL5,CCL7,CCL20,CXCL6,SELE,SELPLG,CXCR4,CH25H,CCL26,TBX21,ASB2,TREM1,MCOLN2,AGTR1,EPHA2,EPHB1,HMGB2,NR4A1,LOX,PDGFRA,TPBG,CXCL14,BMPR1B,C3AR1,DPP4,TYMP,EGR2,FGF7,FPR3,L1CAM,LGALS9,PLAUR,PTAFR,NECTIN1,VEGFC,FOSL1,CDK5R1,NRG3,VSTM2L,DUSP1,SH2B3,RBM15,ZC3H12A,NES,ADCYAP1,ASIP,NPPA,PYY,WNT7B,APLN,RAPGEF2,NMU,NPFFR2,GAL,ARRDC3,PACRG,ADM5 |
| BP | GO:0032649 | regulation of interferon-gamma production | -9.73 | BCL3,CD14,CCR7,DDIT3,F2RL1,GATA3,IRF8,IL1B,IL1R1,IL12A,IL12RB2,INHBA,LGALS9,TLR4,TNFSF4,SLC7A5,IL1RL1,ISG15,BTN3A1,NOD2,ZC3H12A,PDCD1LG2,IFNL1,ADCY7,BCL6,BST2,EPHA2,HFE,IL6,NFKB1,OAS1,OAS3,SRGN,PTGER4,RELB,THBS1,TNFAIP3,CD83,KLF4,TBX21,ERRFI1,CACTIN,DHX58,CD36,HLA-E,HSPB1,OAS2,PTAFR,DDX58,IFIH1,IL33,MAPK13,EGR1,SPHK1,GBP5,SERPINE1,IRF1,SAA1,IL17B,PER1 |
| BP | GO:0010942 | positive regulation of cell death | -9.62 | PARP1,ALDH1A3,ATF3,BCL6,BMPR1B,CALCR,CAMK2A,CD36,CYP1B1,DDIT3,DUSP6,EGR1,FOS,NR4A1,HOXA5,HPGD,HPR,IFIT2,IL6,IL12A,INHBA,ITGA4,ITGAM,JUN,LGALS9,MMP9,MT3,GADD45B,PIK3CD,PMAIP1,PTGIS,PTGS2,CCL2,CCL5,SFRP2,SKIL,THBS1,TLR4,TNFRSF1B,TOP2A,PHLDA2,FOSL1,KLF11,TNFRSF10B,CDK5R1,SQSTM1,RAPGEF2,ANKRD1,DKKL1,G0S2,GAL,IL20RA,ADCY10,ZC3H12A,SLFN11,OMA1,ACVR1C,PXT1,PCSK9 |
| BP | GO:0009725 | response to hormone | -9.54 | ACAT1,ACTA1,PARP1,AGTR1,ASIP,BMP6,KLF9,CALCR,CAMK2A,CDC6,CCR7,CRY1,CTSS,CYP1B1,EGR1,EGR2,FOS,FOSB,GATA3,GPT,HADH,HMGB2,NR4A1,HES1,HSD11B2,IL6,INHBA,LMO2,LOX,MAOB,MT3,NFKB1,NR4A2,PAPPA,PCK1,PDK4,PTAFR,PTGER4,PTGFR,PTGS2,RPE65,RXRG,SGK1,SLC5A5,SSTR1,STC1,THBS1,TRH,TNFSF4,UGCG,WNT7B,TRIM25,NR4A3,CSRP3,FOSL1,BCAR3,STC2,NR1D1,SORBS1,GPR83,SLC2A8,DHH,GAL,ERRFI1,RXFP1,ACVR1C,HAPLN3,PCSK9,ACHE,ADCY5,BLM,CASQ2,CAV3,CD36,CDH1,DRD1,GPD1,HRH1,HRH2,HTR7,ICAM1,ITGA4,JUN,PDGFRA,MAPK13,SLC8A1,TLR4,KLF11,CDK5R1,RAPGEF2,HCN4,DDX58,NSG2,NOD2,IFIH1,MMP9,NFKBIA,TNFAIP3,CYP1A1,IL1B,CCL2,CCL5,HAND2,ZC3H12A |
| BP | GO:0048511 | rhythmic process | -9.26 | BMPR1B,KLF9,CDK1,CRY1,CYP1B1,DBP,EGR1,HAS2,HCRTR2,HLF,HTR7,INHBA,KCND2,NFKB2,PDGFRA,PER1,PTPRN,PTX3,RELB,RPE65,KLF10,TOP2A,BHLHE40,CDK5R1,PER2,ADAMTS1,NR1D1,GPR176,NOCT,DTL,BHLHE41,SPSB4,CAVIN3,ZPBP2,CIART,GATA3,HES1,SNAI1,HAND2,PDE6B,FBXL22 |

| **Table S5. KEGG pathway analysis of 1448 DEGs in GSE150392** | | | |
| --- | --- | --- | --- |
| **Term** | **Description** | **LogP** | **Symbols** |
| hsa04668 | TNF signaling pathway | -18.34 | BIRC3,BCL3,MAP3K8,CSF1,FOS,CXCL1,CXCL2,CXCL3,ICAM1,IL1B,IL6,IRF1,JUN,JUNB,LIF,MMP9,NFKB1,NFKBIA,PIK3CD,MAPK13,PTGS2,CCL2,CCL5,CCL20,CXCL6,SELE,TNFAIP3,TNFRSF1B,TRAF1,VEGFC,NOD2,MLKL,PARP1,BCL2A1,CD14,IL1R1,CXCL8,GADD45B,NFKB2,RELB,TLR4,TRIM25,DDX58,CARD11,FOSB,CCL7,FOSL1,IL17B,CAMK2A,CD36,CYP1A1,DDIT3,HSPA6,IL12A,IRF7,NCF2,NFATC2,PLCB2,RXRG,TNFRSF10B,NCF1,ITGAM,CLK4,ATP6V1G2,CTSK,IL11,GBP3,OAS1,OAS2,OAS3,TRPV2,CARD6,GBP5,CALCR,SQSTM1,IRF8,ITGA5,ITGA4,SERPINE1,HSPB1,EGR2,CXCR1,IGSF5,BMPR1B,CAV3,DUSP1,ADCY5,ADCY7,CDC20,CDKN2C,EGR1,HLA-E,IL15RA,CCNB2,ANAPC16,GATA3,IL12RB2,TBX21,GNG7,PDGFRA,PTGER4,TAP1,CXCR4,ARHGEF28,RLN2,RXFP1,BIRC5,IFIH1,MICB,ADH1B,C3AR1,BST2,CDK1,CUL4B,SAMHD1,CD44,MYL2,MYL9,MYL7 |
| hsa04060 | Cytokine-cytokine receptor interaction | -16.78 | BMP6,BMPR1B,CCR7,CNTF,CSF1,CSF2RB,CTF1,GDF9,CXCL1,CXCL2,CXCL3,IL1B,IL1R1,IL6,IL7R,CXCL8,CXCR1,IL11,IL12A,IL12RB2,IL15RA,TNFRSF9,INHBA,LIF,OSM,CCL2,CCL5,CCL7,CCL20,CXCL6,TNFRSF1B,TNFSF4,CXCR4,TNFSF9,TNFRSF10B,IL1RL1,OSMR,GDF15,CXCL14,CCL26,IL24,CLCF1,IL17B,IL20RA,INHBE,RELT,IL33,ACVR1C,IFNL1,ADCY5,ADCY7,GNG7,NFKB1,NFKBIA,PIK3CD,PLCB2,NCF1 |
| hsa04260 | Cardiac muscle contraction | -7.33 | ACTC1,ATP2A3,CASQ2,COX6A2,COX6C,COX7B,COX7C,HRC,MYL2,MYL4,SLC8A1,TNNC1,TNNI3,TNNT2,TRDN,CACNG4,SLC9A7,ADCY5,ADCY7,DES,ITGA4,ITGA5,SGCA,IL6,AGTR1,CAMK2A,PLCB2,PPP1R1A,MAPK13 |
| hsa05164 | Influenza A | -7.10 | ICAM1,IL1B,IL6,CXCL8,IL12A,IRF7,MX1,MX2,NFKB1,NFKBIA,OAS1,OAS2,OAS3,PIK3CD,PLG,CCL2,CCL5,TLR4,TRIM25,TNFRSF10B,DDX58,IFIH1,IL33,RSAD2,FOS,HSPA6,JUN,TNFAIP3,RAB9B,AGTR1,CFB,C3AR1,MAPK13,RPL3L,ISG15,ACE2,CD44,HLA-E,HES1,GADD45B,NFKB2,RELB,TAP1,DHX58 |
| hsa05202 | Transcriptional misregulation in cancer | -6.11 | BIRC3,BCL2A1,BCL6,CD14,CDKN2C,DDIT3,DUSP6,ETV4,HHEX,HPGD,IL6,CXCL8,ITGAM,LMO2,MMP9,GADD45B,NFKB1,RXRG,SPINT1,TRAF1,NR4A3,PER2,SIX4,NFKBIZ |
| hsa05143 | African trypanosomiasis | -5.92 | F2RL1,HPR,ICAM1,IL1B,IL6,IL12A,NPPA,PLCB2,SELE,APOL1,AGTR1,EGR1,CXCL8,JUN,NFKB1,SERPINE1,PIK3CD,MAPK13,CCL2,VEGFC,CD36,THBS1,TLR4 |
| hsa04010 | MAPK signaling pathway | -5.61 | CD14,MAP3K8,CSF1,DDIT3,DUSP1,DUSP4,DUSP6,EPHA2,FGF7,FGFR3,FOS,NR4A1,HSPA6,HSPB1,IL1B,IL1R1,JUN,GADD45B,NFKB1,NFKB2,NTF4,PDGFRA,PLA2G4A,MAPK13,RASGRF2,RELB,VEGFC,RAPGEF2,CACNG4,DUSP16 |
| hsa04080 | Neuroactive ligand-receptor interaction | -5.46 | ADCYAP1,AGTR1,C3AR1,CALCR,CCKAR,DRD1,F2RL1,FPR3,GABRA4,GABRR2,HCRTR2,HRH1,HRH2,HTR7,PLG,PYY,PTAFR,PTGER4,PTGFR,RLN2,SSTR1,TRH,VIPR1,APLN,NMU,NPFFR2,GPR83,LPAR3,GAL,RXFP1,ADM2,SPX,LYPD6,GPR156 |
| hsa04020 | Calcium signaling pathway | -5.42 | ADCY7,AGTR1,ATP2A3,CAMK2A,CASQ1,CASQ2,CCKAR,DRD1,FGF7,FGFR3,HRH1,HRC,HRH2,HTR7,PDE1A,PDGFRA,PLCB2,PTAFR,PTGFR,SLC8A1,TNNC1,VEGFC,CXCR4,SPHK1,TRDN,MCOLN2 |
| hsa05415 | Diabetic cardiomyopathy | -5.19 | PARP1,AGTR1,ATP2A3,ATP5MC3,ATP5PF,CAMK2A,CD36,COX6A2,COX6C,COX7B,COX7C,MMP9,NCF2,NDUFB1,NDUFS6,NFKB1,PDK4,PIK3CD,PLCB2,MAPK13,TNNI3,GFPT2,NCF1,CYP1A1,CYP1B1,EPHX2,FOS,JUN,NFKBIA,SLC26A9,DDIT3,IL1B,IL6,CXCL8 |
| hsa04913 | Ovarian steroidogenesis | -4.57 | ADCY5,ADCY7,BMP6,CYP1A1,CYP1B1,CYP19A1,PLA2G4A,PTGS2,ACOT2,ACOT1,FABP4,NPPA,PIK3CD,PTGS1 |
| hsa04210 | Apoptosis | -4.55 | PARP1,BIRC3,BIRC5,BCL2A1,CSF2RB,CTSK,CTSS,DDIT3,FOS,JUN,GADD45B,NFKB1,NFKBIA,PIK3CD,PMAIP1,TRAF1,TNFRSF10B,ADCY5,ADCY7,AGTR1,CAMK2A,CDH1,FGF7,FGFR3,GNG7,HES1,IL6,IL7R,CXCL8,IL12A,IL12RB2,IL15RA,MMP9,NFKB2,PDGFRA,PLCB2,PTGER4,PTGS2,RXRG,VEGFC,WNT7B,CXCR4,LPAR3 |
| hsa04750 | Inflammatory mediator regulation of TRP channels | -4.49 | ADCY5,ADCY7,CAMK2A,F2RL1,HRH1,IL1B,IL1R1,PIK3CD,PLA2G4A,PLCB2,MAPK13,PTGER4,TRPV2,ASIC4,AGTR1,CXCL8,CXCR1,PDGFRA,PTGFR,SPHK1,LPAR3,DGKK |
| hsa05203 | Viral carcinogenesis | -4.18 | CDK1,CDC20,EGR2,H2BC5,HLA-E,IRF7,JUN,NFKB1,NFKB2,NFKBIA,PIK3CD,PMAIP1,SP100,TRAF1,H2BC8,H2BC7,H2BC6,H4C3,H4C2,GTF2A1L,H2BC18,FPR3,ITGAM,NCF2,PLCB2,MAPK13,SELPLG,TLR4,H2AC20,NCF1 |
| hsa00350 | Tyrosine metabolism | -4.17 | ADH1B,AOC2,MAOB,PNMT,TPO,TYRP1,AOC3,IL4I1,ALAS2,AMT,PGAM2,DPYS,GAD1,SMOX |
| hsa04670 | Leukocyte transendothelial migration | -3.77 | ICAM1,ITGA4,ITGAM,MMP9,MYL2,NCF2,PIK3CD,MAPK13,THY1,CXCR4,MYL9,CLDN14,MYL7,NCF1 |
| hsa04727 | GABAergic synapse | -3.69 | ADCY5,ADCY7,GABRA4,GABRR2,GAD1,GNG7,KCNJ6,SLC6A12,HAP1,SLC38A3,SLC38A2,SLC38A5,DRD1,PDE1A,PDE4C,PDE7A,PDE10A |
| hsa04024 | cAMP signaling pathway | -3.68 | ADCY5,ADCY7,ADCYAP1,ATP2A3,CAMK2A,DRD1,FOS,JUN,NFKB1,NFKBIA,NPPA,PDE4C,PIK3CD,FXYD1,SSTR1,TNNI3,HCN4,MYL9,PDE10A,ADCY10,SUCNR1 |
| hsa04630 | JAK-STAT signaling pathway | -3.60 | CNTF,CSF2RB,CTF1,IL6,IL7R,IL11,IL12A,IL12RB2,IL15RA,LIF,OSM,PDGFRA,PIK3CD,OSMR,IL24,IL20RA,IFNL1 |
| hsa04713 | Circadian entrainment | -3.34 | ADCY5,ADCY7,ADCYAP1,CAMK2A,FOS,GNG7,GUCY1A1,KCNJ6,PER1,PLCB2,PER2,ADCY10,JUN,NFATC2,NPPA,PLA2G4A,PTGS2,MYL9,CACNG4,AGTR1,PDE1A,PTGER4 |

| **Table S6. GO analysis of 239 common DEGs in GSE120852** | | | | |
| --- | --- | --- | --- | --- |
| **Category** | **Term** | **Description** | **LogP** | **Symbols** |
| CC | GO:0031012 | extracellular matrix | -25.03 | ANXA1,COL1A2,COL3A1,COL7A1,COL8A2,COL12A1,COL15A1,DPT,ECM2,FBN1,FMOD,ANOS1,LAD1,LUM,MDK,MFAP4,MMP9,OGN,PCOLCE,CXCL12,TGFBI,TIMP2,COL14A1,FCN3,LRRC17,SULF1,FLRT2,MXRA5,ABI3BP,SCARA3,ASPN,HAPLN2,SMOC2,ITIH5,COLEC12,KAZALD1,HMCN1,CTHRC1,CCDC80,FREM1,SSC5D,CD4,CTSK,TLL2,C1QTNF2,C1QTNF7,C1QTNF9,MYOT,TUBA3E,TUBA3D,CASQ1,CES1,MXRA8,CHRDL1,DPYSL3,MYO7B,RAC2,FSCN1,TPPP3 |
| BP | GO:0030155 | regulation of cell adhesion | -11.77 | ANXA1,APOD,BMP6,RUNX3,CD4,DAB2,ECM2,EFNB3,HLA-DMA,HLA-DPA1,HLA-DPB1,HLA-DRA,HLA-DRB5,ITGA4,MDK,PIK3CG,PLAU,PTPRC,RAC2,CCL5,CX3CL1,CXCL12,TGFBI,THY1,PLXNC1,TNFSF13B,CD300A,ABI3BP,TNFRSF21,NLRP3,CCDC80,PRDM1,CST7,IL33,CSF1R,EGR1,LUM,OAS1,OAS2,OAS3,BTN3A1,SULF1,CSF2RB,FBN1,LTF,LRRC17,PTN |
| BP | GO:0006935 | chemotaxis | -10.82 | ANXA1,CSF1R,CX3CR1,EFNB3,CXCR2,ANOS1,LSP1,MDK,ENPP2,PIK3CG,PLAU,PTN,NECTIN1,RAC2,CCL5,CX3CL1,CXCL12,CH25H,MYOT,CXCL14,PLXNC1,PALLD,FLRT2,CKLF,CASQ1,ITGA4,PLPPR4,TENM4,DOCK10,NRK,GARIN1A,APOD,TNFRSF21,CTHRC1,COL15A1,ANTXR1 |
| CC | GO:0098552 | side of membrane | -10.31 | ANXA1,CD4,CD8A,CSF2RB,CTSK,CX3CR1,ACE,FOLR2,HLA-DPA1,HLA-DPB1,HLA-DRA,HLA-DRB5,IL2RG,CXCR2,ITGA4,ITGAL,NT5E,PTPRC,CXCL12,THY1,FCN3,BTN3A3,BTN3A1,GNG2,GNB4,ANTXR1,CD163L1,C2,LCP2,MFAP4,PRKCB,CCL5,TNFSF13B,TNFRSF21,NLRP3,OAS1,OAS3,COLEC12,GABBR1,MME,SULF1 |
| BP | GO:0098609 | cell-cell adhesion | -10.29 | ANXA1,COL8A2,CX3CR1,DSC1,ITGA4,ITGAL,NINJ2,NT5E,PIK3CG,PTPRC,NECTIN1,RAC2,CCL5,CX3CL1,SELL,THY1,COL14A1,MYOT,CD93,PALLD,TENM4,JAM2,HMCN1,ROPN1B |
| BP | GO:0050900 | leukocyte migration | -8.80 | ANXA1,CX3CR1,CXCR2,ITGA4,ITGAL,MDK,MMP9,PIK3CG,PTN,CCL5,CX3CL1,SELL,CH25H,CKLF,JAM2,CXCL12,CXCL14,LCP2,CD93,CPE,NKG7,NTRK2,SYN2,ABCC5,SLC40A1,SYTL2,PLAU,ACE,APOD,OGN,EGR1 |
| MF | GO:0005539 | glycosaminoglycan binding | -8.65 | DPYSL3,ECM2,FBN1,ANOS1,LTF,MDK,PCOLCE,PTN,PTPRC,SELL,SULF1,HAPLN2,SMOC2,NLRP3,CCDC80,CBS,GNMT |
| BP | GO:0045321 | leukocyte activation | -8.20 | ANXA1,CD4,CD8A,CX3CR1,EGR1,CXCR2,ITGA4,ITGAL,LCP2,MDK,NKG7,PIK3CG,PRF1,PRKCB,PTPRC,CCL5,CX3CL1,TNFSF13B,BTN3A1,CD93,DOCK10,COL3A1,CSF1R,MMP9,RUNX3,ACE,LRRC17,SLC40A1,BLM |
| MF | GO:0005518 | collagen binding | -8.17 | CTSK,ECM2,LUM,MMP9,PCOLCE,TGFBI,COL14A1,ASPN,ANTXR1 |
| BP | GO:0002685 | regulation of leukocyte migration | -8.14 | ANXA1,APOD,CSF1R,CX3CR1,ITGA4,MDK,PTN,RAC2,CCL5,CX3CL1,CXCL12,THY1,CD300A,JAM2,DAB2,MMP9,ENPP2,PIK3CG,PLAU,PTPRC,SMOC2,CXCL14,CKLF,AVPR1A,ACE,ARRDC3,CTHRC1,CXCR2,LTF,CD4,EFNB3,NECTIN1,TNFSF13B |
| BP | GO:0002252 | immune effector process | -8.10 | C2,CD8A,CSF2RB,CX3CR1,ACE,HLA-DMA,HLA-DPA1,HLA-DPB1,HLA-DRA,HLA-DRB5,ITGAL,MDK,MFAP4,NKG7,PIK3CG,PRF1,FCN3,BTN3A3,DOCK10,CD4,ACP5,CTSK,DAB2,FMOD,CXCR2,LUM,OGN,CST7,GNLY,SIDT1,ARRDC3,GNB4,ANXA1,PRDM1,PRKCB,BTN3A1,TNFRSF21,CPE,DPYSL3,GABBR1,MME,SYN2,SCGN,SLC40A1,SYTL2,IL33,GALNT15,ITGA4,AVPR1A,LTF,RAC2,COLEC12,COL7A1,TGFBI,GOLGA8A,SULF1,B4GALNT3,CHSY3,FOLR2,GIPR,GNMT,GRB14,NTRK2,ABCC5,ANTXR1 |
| MF | GO:0005178 | integrin binding | -7.96 | COL3A1,ECM2,FBN1,ITGA4,ITGAL,PTN,CX3CL1,CXCL12,TGFBI,THY1,ITGBL1,JAM2,ANXA1,CPE,LAD1,NECTIN1,FSCN1,PALLD,TENM4,ARHGAP18,CHRDL1,PLAU,FREM1,ANTXR1 |
| CC | GO:0005604 | basement membrane | -7.94 | COL7A1,COL8A2,COL15A1,FBN1,LAD1,TGFBI,SMOC2,HMCN1,CCDC80,FREM1 |
| BP | GO:0002683 | negative regulation of immune system process | -7.41 | ANXA1,APOD,RUNX3,COL3A1,CX3CR1,FBN1,LTF,MDK,OAS1,OAS3,PTPRC,CX3CL1,CXCL12,THY1,CST7,LRRC17,CD300A,TNFRSF21,DPYSL3,SULF1,ARRDC3,GIPR,PIK3CG,PLAU |
| BP | GO:0050727 | regulation of inflammatory response | -7.12 | ACP5,ANXA1,AOAH,ACE,ESR1,MDK,MMP9,NKG7,NT5E,PIK3CG,PTPRC,CCL5,CX3CL1,CST7,IL33,NLRP3,BCL6B,OAS1,OAS3,FCN3,LTF,PLAU |
| BP | GO:0048525 | negative regulation of viral process | -7.05 | LTF,MX1,OAS1,OAS2,OAS3,CCL5,FCN3,OASL,CH25H,CD4,MX2,PRF1,PTPRC,CXCL12,IFI44L,NLRP3,ACP5,ANXA1,APOD,CX3CR1,CX3CL1,TNFRSF21,SSC5D,CARD17,IL33,SIDT1,STRBP,C2,CSF2RB,ACE,GNLY,COLEC12,HMCN1,CCDC80,CSF1R,EGR1,CCDC3,COL3A1,CD300A,BMP6 |
| BP | GO:0032103 | positive regulation of response to external stimulus | -7.04 | BMP6,CSF1R,CX3CR1,ACE,MDK,NKG7,PIK3CG,PLAU,PTN,RAC2,CCL5,CX3CL1,CXCL12,FCN3,OASL,SMOC2,IL33,NLRP3,ANXA1,FOLR2,CXCR2,ITGAL,AOC3,DOCK10 |
| BP | GO:0019221 | cytokine-mediated signaling pathway | -7.00 | CD4,CSF1R,CSF2RB,CX3CR1,EGR1,IL2RG,CXCR2,MX1,OAS1,OAS2,CCL5,CX3CL1,CXCL12,OASL,TNFSF13B,IL33,DPYSL3,HLA-DPA1,ITGA4,LSP1,MME,TNFRSF21,CHRDL1,HLA-DRA |
| CC | GO:0030934 | anchoring collagen complex | -6.21 | COL7A1,COL12A1,COL14A1,ITGA4,MMP9,APLNR,TENM4,FBN1,FRZB,DACT1,CTHRC1 |
| BP | GO:0046886 | positive regulation of hormone biosynthetic process | -6.20 | BMP6,DAB2,EGR1,POR,ANXA1,AVPR1A,CES1,ACE,GABBR1,GIPR,KCNJ11,OAS2,PRKCB,RAC2,CCL5,CXCL12,SLC6A1,CD300A,CCDC3,APOD,PIK3CG,C1QTNF2,CTSK,FRZB,MDK,CPE,ESR1,GNMT,PLIN2 |

| **Table S7. KEGG pathway analysis of 239 common DEGs in GSE120852** | | | |
| --- | --- | --- | --- |
| **Term** | **Description** | **LogP** | **Symbols** |
| hsa05164 | Influenza A | -9.54 | HLA-DMA,HLA-DPA1,HLA-DPB1,HLA-DRA,HLA-DRB5,MX1,MX2,OAS1,OAS2,OAS3,PRKCB,CCL5,IL33,NLRP3,ACP5,CTSK,ITGAL,CXCL12,TNFSF13B,CD4,CD8A,ITGA4,PTPRC,NECTIN1,SELL,JAM2,CSF1R,MME,PRF1,RAC2,CPE,FCGR3B,RUNX3,IL2RG,C2,COLEC12,TUBA3E,TUBA3D,EGR1,PIK3CG,LSP1,ZNF682 |
| hsa04060 | Cytokine-cytokine receptor interaction | -7.35 | BMP6,CD4,CSF1R,CSF2RB,CX3CR1,IL2RG,CXCR2,CCL5,CX3CL1,CXCL12,IL32,CXCL14,TNFSF13B,TNFRSF21,IL33 |
| hsa04062 | Chemokine signaling pathway | -6.05 | CX3CR1,CXCR2,PIK3CG,PRKCB,RAC2,CCL5,CX3CL1,CXCL12,CXCL14,GNG2,GNB4,GABBR1,SLC6A1,CSF1R,CSF2RB,ESR1,GSTM5,IL2RG,MMP9,APLNR,EGR1,COL1A2,ITGA4,NTRK2,CD4 |
| hsa04974 | Protein digestion and absorption | -5.52 | COL1A2,COL3A1,COL7A1,COL8A2,COL12A1,COL15A1,MME,COL14A1 |
| hsa04670 | Leukocyte transendothelial migration | -5.19 | ITGA4,ITGAL,MMP9,PRKCB,RAC2,CXCL12,THY1,JAM2,FCGR3B,LCP2,PRF1,PTPRC,CSF1R |
| hsa05171 | Coronavirus disease - COVID-19 | -3.73 | C2,ACE,MX1,MX2,OAS1,OAS2,OAS3,PRKCB,NLRP3,IL2RG,CCL5,ANTXR1 |
| hsa05340 | Primary immunodeficiency | -3.51 | CD4,CD8A,IL2RG,PTPRC,ITGA4,LCP2,RAC2,NLRP3 |
| hsa04210 | Apoptosis | -2.94 | BCL2A1,CSF2RB,CTSK,PRF1,TUBA3E,TUBA3D,PRKCB |
| hsa04064 | NF-kappa B signaling pathway | -2.69 | BCL2A1,PLAU,PRKCB,CXCL12,TNFSF13B |
| hsa04929 | GnRH secretion | -2.66 | GABBR1,KCNJ11,KCNN3,PRKCB |
| hsa00260 | Glycine, serine and threonine metabolism | -2.32 | CBS,AOC3,GNMT |
| hsa04380 | Osteoclast differentiation | -2.31 | ACP5,CSF1R,CTSK,FCGR3B,LCP2 |
| hsa04926 | Relaxin signaling pathway | -2.29 | COL1A2,COL3A1,MMP9,GNG2,GNB4,ACE,PRKCB,RAC2,ESR1,LUM,PLAU,EGR1,LCP2,PIK3CG |
| hsa00480 | Glutathione metabolism | -1.87 | GPX3,GSTM5,HPGDS |
| hsa03250 | Viral life cycle - HIV-1 | -1.77 | CD4,MX1,MX2 |
| hsa05221 | Acute myeloid leukemia | -1.70 | BCL2A1,CSF1R,DUSP6,MMP9,PLAU |
| hsa05120 | Epithelial cell signaling in Helicobacter pylori infection | -1.65 | CXCR2,CCL5,JAM2 |
| hsa04512 | ECM-receptor interaction | -1.40 | COL1A2,ITGA4,FREM1 |
| hsa04350 | TGF-beta signaling pathway | -1.33 | BMP6,FBN1,FMOD |

| **Table S8. Different expression miRNAs from GSE104150** | | | | | | | | | | | |
| --- | --- | --- | --- | --- | --- | --- | --- | --- | --- | --- | --- |
| **miRNAs** | **logFC** | **P.Value** | **miRNAs** | **logFC** | **P.Value** | **miRNAs** | **logFC** | **P.Value** | **miRNAs** | **logFC** | **P.Value** |
| hsa-miR-1275 | 5.613954 | 9.07E-13 | hsa-miR-423-5p | 4.332269 | 0.000162 | hsa-miR-6124 | 3.27306 | 0.00398 | hsa-miR-4455 | 3.732704 | 0.024763 |
| hsa-miR-1268a | 5.580839 | 1.9E-12 | hsa-miR-320a | 4.696138 | 0.00023 | hsa-miR-6893-5p | 3.308827 | 0.005062 | hsa-miR-15a-3p | -1.64951 | 0.024943 |
| hsa-miR-6752-5p | 5.500119 | 9.11E-12 | hsa-miR-4485-3p | 4.383549 | 0.000309 | hsa-miR-223-3p | -4.43078 | 0.005264 | hsa-miR-2392 | 2.1575 | 0.025193 |
| hsa-miR-939-5p | 5.352199 | 5.18E-10 | hsa-miR-197-5p | 4.797028 | 0.000424 | hsa-miR-5787 | 3.651188 | 0.006535 | hsa-miR-576-5p | -1.9521 | 0.026626 |
| hsa-miR-4532 | 5.391279 | 3.79E-09 | hsa-miR-4632-5p | 4.16401 | 0.000546 | hsa-miR-3149 | 3.42604 | 0.007141 | hsa-miR-4649-3p | 2.03987 | 0.026849 |
| hsa-miR-494-3p | 5.654365 | 8.02E-09 | hsa-miR-6751-3p | 4.537453 | 0.000674 | hsa-miR-6791-5p | -1.08062 | 0.01035 | hsa-miR-548aq-5p | -2.31229 | 0.027162 |
| hsa-miR-4769-3p | 4.746777 | 1.35E-08 | hsa-miR-4485-5p | 4.321609 | 0.000711 | hsa-miR-16-5p | -3.41725 | 0.011394 | hsa-let-7g-5p | -3.22058 | 0.027241 |
| hsa-miR-3196 | 4.505249 | 1.74E-07 | hsa-miR-486-5p | 1.250614 | 0.000745 | hsa-miR-937-5p | 2.694092 | 0.011917 | hsa-miR-26b-5p | -2.57551 | 0.027294 |
| hsa-miR-6812-5p | 4.090182 | 8.79E-07 | hsa-miR-150-3p | 4.79893 | 0.001281 | hsa-miR-4741 | 2.804844 | 0.01196 | hsa-miR-6875-5p | 2.989706 | 0.029335 |
| hsa-miR-4459 | 1.881585 | 1.19E-06 | hsa-miR-186-3p | -2.5567 | 0.001422 | hsa-miR-4484 | 3.415072 | 0.013516 | hsa-miR-548aj-3p | -2.75199 | 0.029512 |
| hsa-miR-4433a-3p | -1.31491 | 2.69E-06 | hsa-miR-320e | 3.996785 | 0.001717 | hsa-miR-1229-5p | 3.015224 | 0.013779 | hsa-miR-1225-5p | 2.418617 | 0.030485 |
| hsa-miR-5739 | 4.395119 | 3.52E-06 | hsa-miR-718 | 3.485791 | 0.00174 | hsa-miR-30d-5p | 3.551325 | 0.013867 | hsa-miR-4499 | 2.024386 | 0.030533 |
| hsa-miR-7847-3p | 4.706946 | 4.63E-06 | hsa-let-7a-5p | -4.44835 | 0.001835 | hsa-miR-15a-5p | -3.52477 | 0.014957 | hsa-miR-7641 | 1.991341 | 0.031726 |
| hsa-miR-4687-3p | 1.559843 | 5.73E-06 | hsa-miR-5196-5p | 3.794604 | 0.001862 | hsa-miR-6785-5p | 3.118188 | 0.015833 | hsa-miR-4634 | 2.443698 | 0.035082 |
| hsa-miR-6756-5p | 4.838564 | 8.44E-06 | hsa-miR-320b | 4.161046 | 0.001887 | hsa-miR-6165 | 2.935757 | 0.016937 | hsa-miR-126-3p | -3.15849 | 0.037635 |
| hsa-miR-1268b | 4.659012 | 2.66E-05 | hsa-let-7f-5p | -4.23532 | 0.002244 | hsa-miR-4270 | 2.855122 | 0.01722 | hsa-miR-876-3p | -1.45314 | 0.03961 |
| hsa-miR-8072 | 4.230217 | 3.06E-05 | hsa-miR-4327 | 4.258966 | 0.00239 | hsa-miR-197-3p | 3.164163 | 0.019125 | hsa-miR-181a-5p | 2.754403 | 0.042024 |
| hsa-miR-483-3p | 4.109002 | 6.03E-05 | hsa-miR-762 | 3.47545 | 0.002628 | hsa-miR-23a-5p | -2.04324 | 0.0205 | hsa-miR-671-5p | 1.949159 | 0.042907 |
| hsa-miR-3195 | 4.427765 | 7.47E-05 | hsa-miR-1273g-3p | 1.013485 | 0.003199 | hsa-miR-3141 | 2.566214 | 0.020864 | hsa-miR-142-3p | -2.13346 | 0.043867 |
| hsa-miR-7106-5p | 3.741508 | 9.96E-05 | hsa-miR-451a | -2.4796 | 0.003314 | hsa-miR-580-3p | -2.32157 | 0.023414 | hsa-miR-4271 | 2.420599 | 0.043972 |
| hsa-miR-6775-5p | 3.917595 | 0.000119 | hsa-miR-320d | 3.876913 | 0.003398 | hsa-miR-548d-5p | -2.24142 | 0.023802 | hsa-miR-4419b | 2.563693 | 0.046144 |
| hsa-miR-6850-5p | -1.04718 | 0.000152 | hsa-miR-6769b-5p | 3.000994 | 0.003636 | hsa-miR-320c | 2.824361 | 0.024654 | hsa-miR-4721 | 2.27674 | 0.047444 |

| **Table S9.**   **Intersecting miRNAs explored from GSE104150 and Targetscan** | | | | | | | |
| --- | --- | --- | --- | --- | --- | --- | --- |
| **Gene** | **miRNA** | **Gene** | **miRNA** | **Gene** | **miRNA** | **Gene** | **miRNA** |
| OAS1 | hsa-miR-1225-5p | OAS2 | hsa-miR-2392 | OAS2 | hsa-miR-5196-5p | OAS2 | hsa-miR-6785-5p |
| OAS1 | hsa-miR-1229-5p | OAS2 | hsa-miR-23a-5p | OAS2 | hsa-miR-548aq-5p | OAS2 | hsa-miR-6812-5p |
| OAS1 | hsa-miR-15a-3p | OAS2 | hsa-miR-26b-5p | OAS2 | hsa-miR-548d-5p | OAS2 | hsa-miR-6850-5p |
| OAS1 | hsa-miR-4270 | OAS2 | hsa-miR-3149 | OAS2 | hsa-miR-576-5p | OAS2 | hsa-miR-6893-5p |
| OAS1 | hsa-miR-6751-3p | OAS2 | hsa-miR-4433a-3p | OAS2 | hsa-miR-5787 | OAS2 | hsa-miR-7106-5p |
| OAS1 | hsa-miR-7106-5p | OAS2 | hsa-miR-4459 | OAS2 | hsa-miR-580-3p | OAS2 | hsa-miR-7641 |
| OAS2 | hsa-miR-1225-5p | OAS2 | hsa-miR-4484 | OAS2 | hsa-miR-6124 | OAS3 | hsa-miR-1273g-3p |
| OAS2 | hsa-miR-1229-5p | OAS2 | hsa-miR-4499 | OAS2 | hsa-miR-671-5p | OAS3 | hsa-miR-197-3p |
| OAS2 | hsa-miR-1275 | OAS2 | hsa-miR-4632-5p | OAS2 | hsa-miR-6751-3p | OAS3 | hsa-miR-23a-5p |
| OAS2 | hsa-miR-181a-5p | OAS2 | hsa-miR-4721 | OAS2 | hsa-miR-6752-5p | OAS3 | hsa-miR-3149 |
| OAS2 | hsa-miR-197-5p | OAS2 | hsa-miR-4769-3p | OAS2 | hsa-miR-6756-5p | OASL | hsa-miR-1273g-3p |
